# Impact of clinical decision support software on empirical antibiotic prescribing and patient outcomes: a systematic review and meta-analysis

**DOI:** 10.1136/bmjopen-2025-099100

**Published:** 2025-11-27

**Authors:** Christopher Hatton, Samuel Quarton, Alana Livesey, Bushra Ali Alenazi, Charlotte Jeff, Elizabeth Sapey

**Affiliations:** 1 National Institute for Health and Care Research (NIHR) Midlands Patient Safety Research Collaboration, University of Birmingham, Birmingham, England, UK; 2 Department of Inflammation & Ageing, School of Infection, Inflammation and Immunology, College of Medicine and Health, University of Birmingham, Birmingham, England, UK; 3 National Institute for Health and Care Research (NIHR) Birmingham Biomedical Research Unit, Birmingham, England, UK; 4 Respiratory Care Department, College of Applied Medical Sciences Jubail, Imam Abdulrahman Bin Faisal University, Dammam, Saudi Arabia

**Keywords:** Antibiotics, Health informatics, Clinical Decision-Making

## Abstract

**Abstract:**

**Objectives:**

To identify Clinical Decision Support Software (CDSS) that have been implemented in hospital which aim to influence empirical antibiotic prescribing, and to establish their impact on antibiotic prescribing and patient outcomes.

**Design:**

Systematic review & meta-analysis.

**Data sources:**

MEDLINE, Cochrane Central Register of Controlled Trials and Embase were searched from their inception to February 2024.

**Eligibility criteria:**

Studies evaluating the impact of digital CDSS with the primary purpose of influencing initial empirical antibiotic prescribing for patients with acute infection in hospital.

**Data extraction and synthesis:**

Study characteristics, intervention characteristics and outcome data were extracted independently by two reviewers. Outcomes were grouped into four domains including clinical outcomes (mortality, length of stay, readmission rates), antibiotic appropriateness (guideline adherence, coverage of causative organism), antimicrobial stewardship and health economics. Risk of bias assessment was conducted using Risk of Bias In Non-randomised Studies - of Interventions for non-randomised studies and Cochrane Risk of Bias 2 for randomised studies. Outcome data with sufficient reporting and homogeneity were synthesised quantitatively using a random-effects meta-analysis; other outcomes were synthesised qualitatively.

**Results:**

15 full texts met the eligibility criteria after screening 7984 unique studies. Low-quality evidence suggested that implementation of CDSS was associated with lower mortality (OR 0.76, 95% CI 0.57 to 1.01) and improved adherence to antibiotic prescribing guidelines (OR 1.75, 95% CI 1.26 to 2.43). No change in length of stay or readmission rates were observed. Coverage of the causative organism was similar after CDSS implementation (OR 1.26, 95% CI 0.97 to 1.63). High-quality evidence supported the association between CDSS implementation and reduced broad-spectrum antibiotic prescribing.

**Conclusions:**

CDSS can be used to reduce the unnecessary prescribing of broad-spectrum antibiotics. Further high-quality studies are required to establish whether their implementation also results in improvements in other outcomes.

**PROSPERO registration number:**

CRD42024501185.

**STRENGTHS AND LIMITATIONS OF THIS STUDY**

- A comprehensive search strategy with broad search terms across multiple databases was used.
- A wide range of outcomes were extracted to capture the impact of Clinical Decision Support Software across several domains.
- Risk of bias was assessed systematically and rigorously using the Risk of Bias In Non-randomised Studies - of Interventions tool and Cochrane Risk of Bias 2 tool.
- Use of broad inclusion criteria increased heterogeneity among included studies.

## Background

 Acute bacterial infection is one of the most common causes of admission to hospital and is a major cause of morbidity and mortality. Antibiotics are a key element of effective treatment and comprise around 10% of all medication prescribed in hospital.^1^ However, their effectiveness is threatened by bacterial antimicrobial resistance (AMR), and it is estimated that several million people per year could die secondary to AMR by 2050 without action.^2^ Excessive and inappropriate prescribing of antibiotics contributes to bacterial AMR, and effective antimicrobial stewardship is rightly a priority of authorities across the world.^3^

In clinical practice, a significant proportion of antibiotic prescribing is empirical; this refers to prescribing prior to identification of a causative microorganism. It requires prescribers to balance multiple competing factors including the likely organism(s), individual and population adverse effects secondary to antibiotic prescribing, and the consequences of treatment failure. Many of these factors are uncertain at the time of empirical antibiotic prescribing. They also vary geographically, temporally and based on individual patient factors. Local and national antibiotic prescribing guidelines are available for most infectious syndromes and attempt to provide rational guidance that takes these factors into consideration. However, adherence to antibiotic prescribing guidelines is often suboptimal and overprescribing of broad-spectrum antibiotics is common, which may cause patient harm.^4^ In addition to increased rates of AMR, overprescribing of broad-spectrum antibiotics is also associated with hospital-acquired infections such as *Clostridium difficile.*^5^ Additionally, the extent to which guidelines provide personalised antibiotic prescribing advice is limited.

Clinical Decision Support Software (CDSS) has been suggested as a potential solution to improve guideline adherence, reduce overprescribing of broad-spectrum antibiotics and personalise antibiotic prescribing.^6^ CDSS are defined as software designed to be a direct aid to clinical decision making, where the characteristics of an individual patient are matched to a computerised knowledge base and patient-specific assessments or recommendations are presented to the clinician.^7^ They are diverse and include rules-based systems that use pre-programmed if-else statements to provide an output, and comparatively complex algorithmic systems that use machine learning or other statistical models to provide an output to clinical decision makers.^8^ They have been applied in multiple settings including primary and secondary care, and across a variety of infectious syndromes to improve antibiotic prescribing, and subsequent patient outcomes.^9^

This systematic review aims to assess the impact of CDSS implemented in hospital to influence empirical antibiotic prescribing. Previous systematic reviews have been conducted,^9^,^15^ but there are important limitations and differences to consider. First, many previously reported systematic reviews do not provide a clear definition of CDSS. Second, this is a rapidly advancing area with several new studies published recently. Third, the scope of this systematic review differs from those previously published as here the focus is on CDSS designed to influence initial empirical antibiotic prescribing in hospital.

## Methods

This systematic review is reported in accordance with Preferred Reporting Items for Systematic Reviews and Meta-Analyses guidelines^16^ and was prospectively registered on PROSPERO (CRD42024501185).

### Eligibility criteria

Studies were considered for inclusion if they implemented digital CDSS in hospital which had the primary purpose of influencing initial empirical antibiotic prescribing for patients with acute infection. Study designs eligible for inclusion included randomised controlled trials (RCTs), controlled and uncontrolled before-after studies and interrupted time series. Only studies that evaluated the impact of CDSS implementation, rather than adherence to CDSS recommendations, were eligible for inclusion. This decision was made because results from analyses comparing adherence to non-adherence are likely to be prone to significant bias; there are likely to be reasons for non-adherence in many cases resulting in non-comparable populations. Detailed eligibility criteria and examples of CDSS that were excluded are provided in table 1.

**Table 1:** Eligibility criteria for included studies

	Inclusion criteria	Exclusion criteria
**Population**	Adults admitted to hospital or presenting to an acute unplanned care service with acute infection or presumed acute infection.	Studies in primary care, community or ambulatory care settings.
**Intervention**	Digital CDSS integrated within or linked to the electronic health record with the primary purpose of influencing initial empirical antibiotic prescribing. Where: CDSS is defined as software that is designed to influence clinical decision making by providing patient-specific recommendations or patient-specific assessments based on the characteristics of individual patients. Initial empirical prescription of antibiotics is defined as the initial prescribing of antibiotics for patients with presumed infection prior to confirmed microbiological diagnosis.	CDSS where the primary purpose is not to influence initial empirical antibiotic prescribing. This includes CDSS with the primary aim of: Definitive or prophylactic antibiotic prescribing. Diagnosis and investigation. Antimicrobial stewardship after initial empirical prescribing, that is, de-escalation of antibiotics. Software defined as CDSS but not meeting the pre-specified definition. This includes CDSS where: Output is not patient specific and based on individual patient information. For example, the presentation of generic drug-drug interactions or side effects, or generic guideline information only.
**Comparator**	Usual care and non-electronic CDSS	
**Outcome**	Clinical outcomes—mortality and morbidity (such as length of hospital stay and readmission to hospital). Appropriateness of antibiotics—guideline adherence, clinician judgement, in vitro susceptibility. Antimicrobial stewardship—volume of antibiotics prescribed, antimicrobial resistance, hospital-acquired infection. Health economics—cost of antibiotics/costs of treatment.	Studies that only include qualitative outcomes such as perceptions of using CDSS.
**Study design**	Randomised controlled trials. Non-randomised studies of interventions including before-after studies and interrupted time series.	Studies that had not implemented CDSS within clinical practice. Case studies. Case series with less than 20 patients.

The table provides a summary of the inclusion and exclusion criteria used for assessing eligibility of individual studies.

CDSS, Clinical Decision Support Software.

CDSS was defined in accordance with previous literature as software intended to directly aid in clinical decision making, where the characteristics of individual patients are matched to a computerised knowledge base to generate patient-specific assessments or recommendations that are presented to clinical decision makers.^17^ This definition was operationalised by requiring the use of individual patient data to provide patient-specific output; thus, digital tools that displayed generic guidelines or generic drug information were excluded.

Initial empirical antibiotic prescribing was defined as the initial prescribing of antibiotics for patients with confirmed or suspected infection prior to the availability of microbiology culture results. Studies that evaluated CDSS which influenced definitive and prophylactic antibiotic prescribing were excluded unless results were clearly separable. While the primary aim was to explore initial empirical prescribing, it is possible that some patients included in studies had antibiotics prescribed in the community prior to attendance at hospital.

### Information sources and search strategy

MEDLINE, Cochrane Central Register of Controlled Trials and Embase were searched from their inception to February 2024 to identify relevant original research. Search strategies were adapted for each individual database and full details are provided in online supplemental material 1.

### Selection process

References were imported into Covidence, a web-based software platform that streamlines the production of systematic and other literature reviews.^18^ Title and abstracts were screened for eligibility by two authors independently. Disagreements were discussed, and if consensus was not reached, a final decision was made by an independent third reviewer. Eligible full texts were also screened by two independent reviewers, with disagreements arbitrated by an independent third reviewer.

### Data collection and data items

Study characteristics, intervention characteristics and outcome data were extracted and transcribed onto a pre-designed data extraction table independently by two reviewers. Outcomes were grouped into four categories including clinical outcomes, antibiotic appropriateness, antimicrobial stewardship and health economic outcomes. Given the sparsity of evidence, no pre-specified time range of interest was defined and all analyses relevant to the outcomes were extracted. Studies rarely reported multiple time points, but where this occurred, short-term outcomes (ie, within 30 days or less) were prioritised for inclusion in quantitative synthesis, as they were considered more likely to be related to the intervention than longer-term outcomes. Some studies reported multiple intervention phases, often including a phase where CDSS was implemented alone, and a phase where it was implemented with other antimicrobial stewardship interventions. In these studies, the intervention phase that reflected the implementation of CDSS with the fewest co-interventions was used as the outcome, and the phase with fewest interventions was used as the baseline comparator.

Clinical outcomes included mortality, length of hospital stay and readmission to hospital. Antibiotic appropriateness included outcomes that measured antibiotic prescribing with reference to best practice clinical guidelines or coverage of the eventual causative organism. These outcomes measure antibiotic use at the individual level and capture both underprescribing and overprescribing of antibiotics. Antimicrobial stewardship included outcomes that primarily measured the overall use and overuse of antibiotics, the prevalence of antimicrobial resistant organisms and healthcare-associated infections. These outcomes measure broader processes related to antibiotic prescribing, usually at the institutional and organisational levels. While there is overlap between antibiotic appropriateness and antimicrobial stewardship, these were deemed to capture different aspects of antibiotic prescribing.

### Study risk of bias assessment

Risk of bias assessment was conducted using Risk of Bias in Non-randomised Studies of Interventions for non-randomised studies and Cochrane Risk of Bias 2 for randomised studies independently by two reviewers with disagreements arbitrated by an independent third reviewer if needed.

### Effect measures and synthesis methods

Outcomes with sufficient reporting and homogeneity were synthesised quantitatively using a random-effects meta-analysis with the *metafor* package^19^ in R V.4.3.0^20^ using the restricted maximum likelihood method. Decisions surrounding whether to perform quantitative synthesis for individual outcomes were made qualitatively, rather than using pre-specified thresholds. Generally, outcomes were quantitatively synthesised if they were reported consistently or could be converted to a consistent measure across most studies. All outcomes that were synthesised quantitatively were dichotomous and were either extracted as ORs or converted to ORs using raw values, p values or 95% CIs of included studies using the *metafor* package. Where adjusted and unadjusted results were reported, adjusted results were extracted. Confounding bias is common in studies evaluating complex interventions, particularly non-randomised studies, and therefore adjusted results were thought to be more representative of the true effect of the intervention. Results were presented graphically using a forest plot. Where this was not possible, a narrative synthesis of results is reported. Heterogeneity among studies was assessed using the I^2^ statistic and Q test and was explored qualitatively. Publication bias was assessed visually using funnel plots and quantitatively using Egger’s test where sufficient data existed. The certainty of evidence for each outcome domain was evaluated qualitatively based on the risk of bias assessments, together with the consistency, directness and precision of results.

### Patient and public involvement

No patients or members of the public were directly involved in this study.

## Results

### Study selection

**Figure 1:**
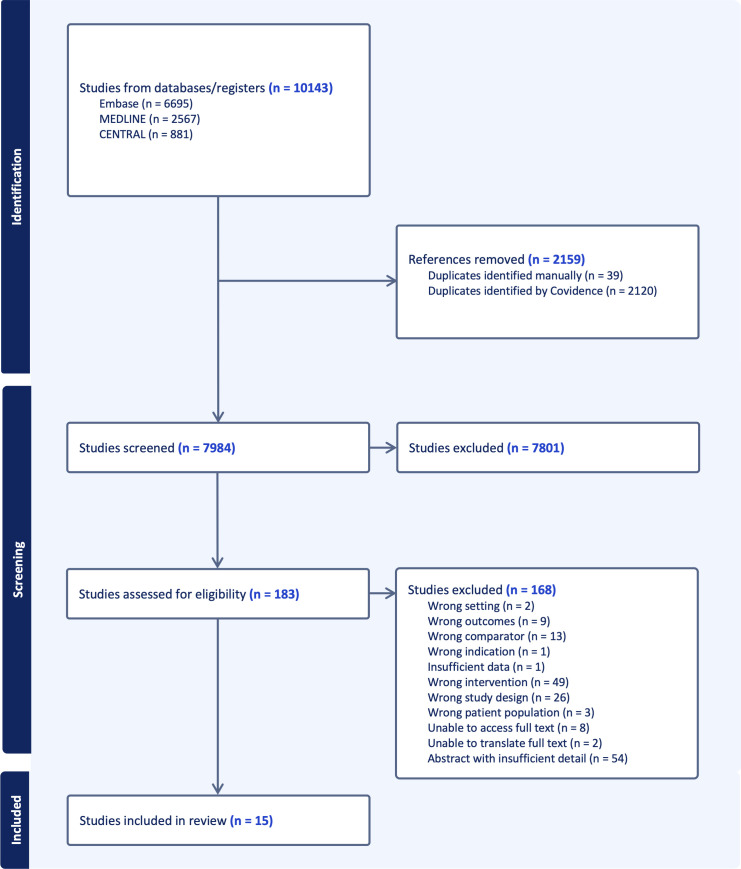
PRISMA flow diagram outlining study selection process. Electronic database search conducted in February 2024. CENTRAL, Cochrane Central Register of Controlled Trials; PRISMA, Preferred Reporting Items for Systematic Reviews and Meta-Analyses.

An overview of the study selection process is provided in figure 1. After removal of duplicates, 7984 unique studies were identified by the search conducted in February 2024. 15 full texts met eligibility criteria after full-text review of 183 articles.^21^,^35^ Several prior systematic reviews have explored a similar question.^9^,^15^ However, there are important differences in the scope and methodology of this systematic review. First, this review uses a definition of CDSS that requires the use of individual patient data to provide a patient-specific output. Software that was labelled as CDSS but that only provided generic outputs, rather than patient specific outputs, was excluded.^36^,^38^ In addition, this review focused on CDSS that influenced initial empirical antibiotic prescribing. There are several examples of CDSS that influence prophylactic or definitive antibiotic prescribing based on known microbial results that were excluded as results were not clearly separable.^39^,^44^ There are also examples of CDSS where the primary aim is antimicrobial stewardship and de-escalation of antibiotics, rather than initial empirical prescribing.^45 46^ Some studies were excluded as the CDSS were not integrated within or linked to the EHR.^47 48^ Others were not implemented into clinical practice^49 50^ or were implemented but without a suitable comparator group.^51^ This list is not exhaustive but highlights how this review differs from other published systematic reviews.

### Study and intervention characteristics

**Figure 2:**
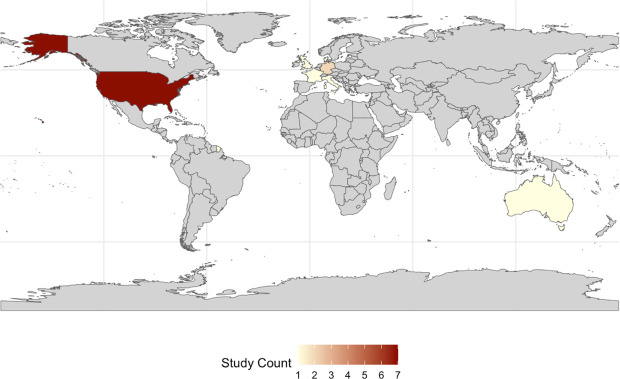
Geographical distribution of studies that evaluated the impact of CDSS that were designed to influence initial empirical antibiotic prescribing in hospital. The frequency of published studies in each country is represented by colour. Grey areas represent countries where no studies have been published. CDSS, Clinical Decision Support Software.

A summary of study characteristics is provided in table 2, and a summary of intervention characteristics is provided in online supplemental material 2. As shown in figure 2, 7 of the 15 included studies were conducted in the USA,^24^,^2729^ 4 were conducted in Europe,^21 22 28 35^ 1 was conducted in Australia,^23^ 1 was conducted in Singapore,^33^ 1 was conducted in Israel^32^ and 1 was conducted internationally across Israel, Germany and Italy.^34^ They were conducted in emergency departments,^23 24 26 27^ acute care wards,^22 25^ intensive care units^25^ and hospital wards.^2129^,^34^ The CDSS evaluated in these 15 studies aimed to improve antibiotic prescribing in pneumonia in 5 studies,^2325^,^27 30^ urinary tract infection (UTI) in 2 studies,^28 31^ skin and soft tissue infection in 1 study^24^ and a mixed cohort of infection in other studies.^2122 29 32^,^35^ The majority of CDSS evaluated in these studies were pre-programmed based on pre-existing medical knowledge and clinical guidelines.^2121 23^,^28 33 35^ Four studies evaluated the effectiveness of algorithmic, or non-knowledge-based, CDSS.^30^,^3234^ 11 distinct CDSS were evaluated across the 15 included studies. Online supplemental material 2 highlights CDSS that were evaluated across more than one study.

**Table 2:** Study characteristics of included studies

Reference	Country	Setting	Infectious syndrome	Study design	Total sample size	Duration of study (months)
Al Bahar *et al*^21^	UK	Hospital	Not specified	Uncontrolled before-after study	Not specified	48 months
Arboe *et al*^22^	Denmark	Acute medical ward	All infections (53% pneumonia, 18% UTI, 8% skin-soft tissue infection)	Uncontrolled before-after study	511 patients	Retrospective study (pre-period)—2 months Prospective study (post-period)—5 months
Buising *et al*^23^	Australia	Emergency department	Pneumonia	Uncontrolled before-after study/interrupted time series	740 patients	Baseline—11 months Academic detailing—8 months CDSS—5 months
Carman *et al*^24^	USA	4 emergency departments	Skin and soft tissue infection (subcutaneous abscess)	Uncontrolled before-after study	873 patients	3 months (12 weeks)
Ciarkowski *et al*^25^	USA	Acute care/ICU pulmonary or hospitalist services	Pneumonia	Uncontrolled before-after study	1021 patients	30 months
Dean *et al*^26^	USA	7 emergency departments	Pneumonia	Controlled before-after study	4760 patients	24 months
Dean *et al*^27^	USA	16 emergency departments	Pneumonia	Pragmatic, stepped-wedge cluster-controlled trial	6848 patients	36 months
Demonchy *et al*^28^	France	3 emergency departments	UTI (cystitis, pyelonephritis, acute prostatitis)	Controlled before-after study	912 patients	7 months (30 weeks)
Evans^29^	USA	Hospital	Not specified	Uncontrolled before-after study	750 patients	12 months
Gohil *et al*^30^	USA	59 private community hospitals	Pneumonia	Cluster-randomised controlled trial	96 451 patients	33 months
Gohil *et al*^31^	USA	59 private community hospitals	UTI	Cluster-randomised controlled trial	127 403 patients	33 months
Leibovici *et al*^32^	Israel	6 internal medicine wards	Mixed—UTI, pneumonia, skin/soft tissue, abdominal, other, ‘non-infectious’	Cluster-randomised controlled trial	1683 patients	Original RCT^34^ was 6 months’ duration. This study looked at 180-day mortality of these patients
Ng *et al*^33^	Singapore	Hospital	52 infective syndromes	Interrupted time series	Not specified	143 months (11 years 11 months)
Paul *et al*^34^	Israel, Italy, Germany	6 internal medicine wards (Israel); 2 gastroenterology, 2 renal, 2 intensive care (Germany); 3 infectious diseases wards (Italy)	Mixed—UTI, pneumonia, skin/soft tissue, abdominal, other, ‘non-infectious’	Cluster-randomised controlled trial	2326 patients	6 months
Röhrig *et al*^35^	Germany	Surgical ICU	Not specified	Uncontrolled before-after study	156 patients	14 months

The table shows the study characteristics of the 15 eligible studies included in this systematic review. Data presented include the country, hospital setting, infectious syndrome, study design, sample size and duration of study.

CDSS, Clinical Decision Support Software; ICU, intensive care unit; RCT, randomised controlled trial; UTI, urinary tract infection.

The majority of studies were non-randomised and study design included uncontrolled before-after studies,^21^,^2529 35^ controlled before-after studies,^26 28^ interrupted time series^33^ and a non-randomised stepped-wedge cluster-controlled trial.^27^ There was variable and incomplete adjustment for potential confounding including disease severity, case-mix and temporal trends across all non-randomised studies. Four studies were cluster-randomised controlled trials, two with cluster assignment at the ward level^32 34^ and two with cluster assignment at the hospital level.^30 31^ Risk of bias assessments for all outcomes in individual studies are provided together with summary plots for each outcome domain in online supplemental material 3 and will be summarised for outcome groups in the following sections.

### Clinical outcomes

**Figure 3:**
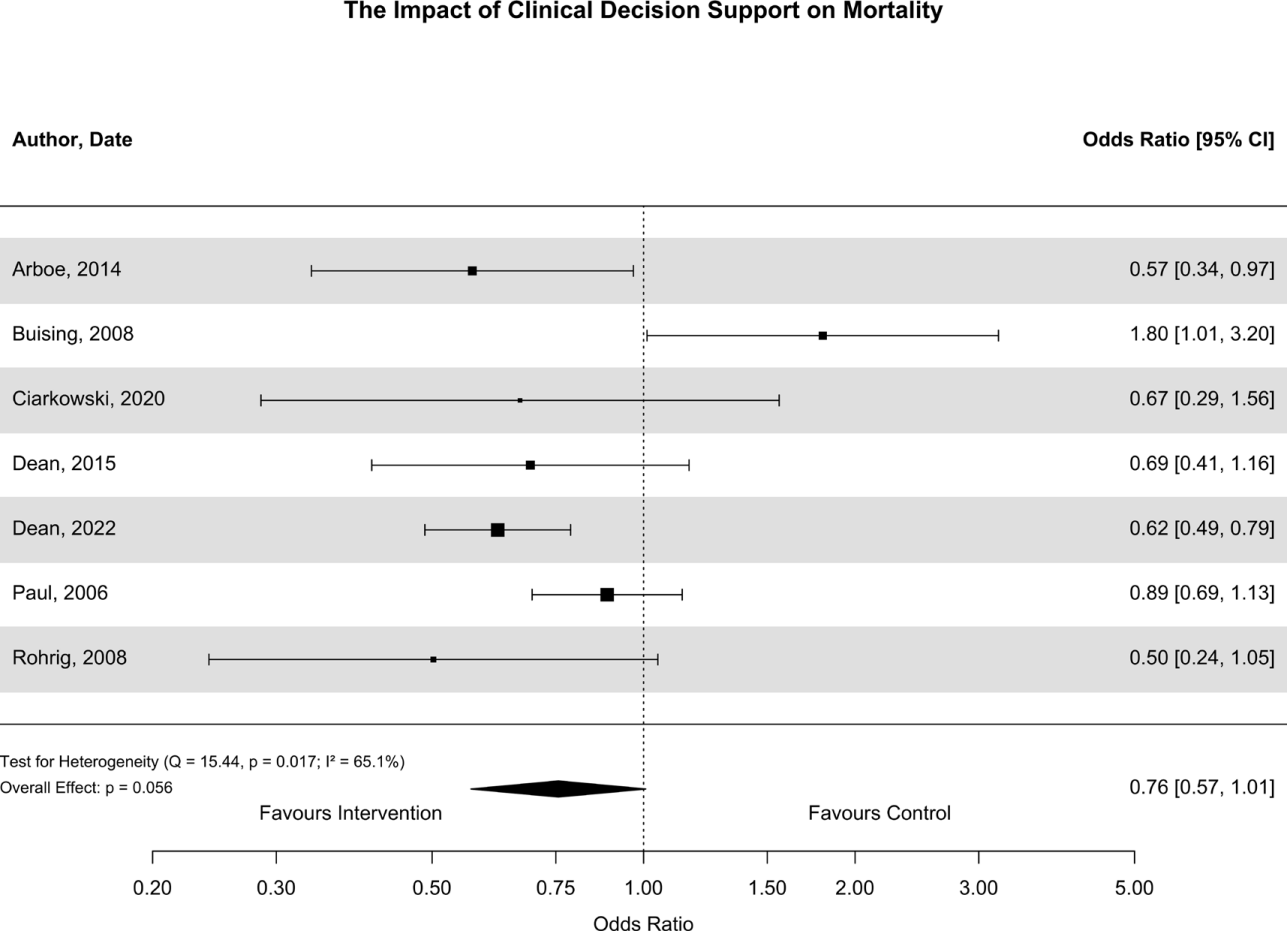
This forest plot summarises the outcomes of studies that reported the impact of CDSS implementation on mortality. Results from adjusted analyses were used to derive ORs and 95% CIs in three studies. Unadjusted ORs were derived from raw data in four studies. Mortality outcome measures included inpatient mortality, ICU mortality and 30-day mortality. Overall, there was a trend towards reduced mortality following CDSS implementation (OR 0.76, 95% CI 0.57 to 1.01, p=0.056). There was significant statistical heterogeneity (I^2^=65.1.9%; Q=15.44, p=0.017). CDSS, Clinical Decision Support Software; ICU, intensive care unit.

The most common clinical outcomes reported included mortality, length of hospital stay, readmission to hospital and time to antibiotics. Mortality was reported in nine studies.^1415 18 19 24^,^27 33^ Seven of these were included in quantitative synthesis as shown in figure 3. One study reporting mortality was excluded as it comprised a subgroup analysis of a study already included in quantitative synthesis with a different follow-up period.^32^ Another study was excluded as the results were derived from a segmented regression analysis of mortality rates which was difficult to combine with results of other studies.^33^ Results of the random effects meta-analysis suggest a trend towards reduced mortality following the implementation of CDSS (OR 0.76, 95% CI 0.57 to 1.01, p=0.056). There was significant statistical heterogeneity across studies (I^2^=65.1%, p=0.017). A substantial proportion of heterogeneity in mortality outcomes arose from a single study, which observed an increased death rate during the CDSS period. However, this result did not adjust for important observed differences in case-mix, which may account for this discrepant finding. Other sources of heterogeneity include differences in study setting, study design, infectious syndrome, CDSS characteristics and outcome measures used. There was no clear and consistent relationship between these features and study results. Measures of mortality included ICU mortality,^35^ 30-day mortality^22 26 27 34^ and inpatient mortality.^25^ One study did not clearly specify the measure of mortality used.^23^ There was no evidence of reporting bias in the funnel plot shown in online supplemental material 4, but the number of included studies is less than the number that is usually recommended to assess for funnel plot asymmetry. Egger’s test did not suggest publication bias existed (p=0.9). Risk of bias for mortality was deemed serious in five out of six non-randomised studies,^22 23 25 26 35^ moderate for one non-randomised study^27^ and high for one cluster RCT.^34^ Of studies included in meta-analysis, unadjusted results were extracted for four studies and adjusted results for three studies.

Heterogeneity in statistical analysis and reporting of other clinical outcomes was too high to justify quantitative synthesis. For example, length of hospital stay was reported as unadjusted summary statistics, as difference-in-difference hazard ratios (HRs) derived from survival analysis and as trend and level changes following segmented regression analysis. Length of hospital stay was reported in eight studies^22 23 25 26 30 31 33 34^ and length of ICU stay in one study.^35^ Most studies that report comparisons before and after the implementation of CDSS do not provide evidence of statistically significant differences in length of hospital stay.^23 25 26 30 31 34^ Readmission rates were similar following the implementation of CDSS in all three studies that reported this outcome.^25^,^27^ Time to antibiotics was reduced following the implementation of CDSS in both studies that reported this outcome by a median difference of 29 min in one study,^23^ and a mean difference of 8.5 min in another study.^27^ A table summarising the results of all studies reporting clinical outcomes is provided in online supplemental material 5. Risk of bias for other clinical outcomes was mixed and is reported individually for each study in online supplemental material 3.

### Antibiotic appropriateness

**Figure 4:**
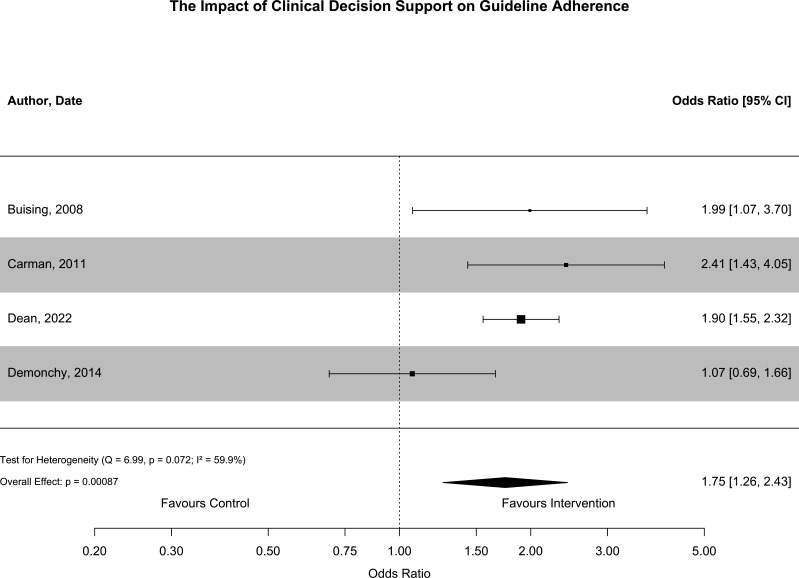
This forest plot summarises the outcomes of studies that reported the impact of CDSS implementation on guideline adherence. Results from adjusted analyses were used to derive OR and 95% CIs for all studies. Guideline adherence improved following the implementation of CDSS (OR 1.75, 95% CI 1.26 to 2.43). There was significant statistical heterogeneity (I^2^=59.9%; Q=6.99, p=0.07). CDSS, Clinical Decision Support Software.

Appropriateness of antibiotic prescribing was evaluated in eight studies.^22^,^2427^ Seven of these were included in quantitative synthesis; one study was excluded because there was limited reporting of how the outcome ‘adequate therapy’ was derived.^35^ Appropriateness was based on guideline adherence in four of these studies^23 24 27 28^ and based on coverage of the causative organism in three studies.^22 29 34^ These outcomes were synthesised in separate meta-analyses and results are provided in figures4  5.

**Figure 5:**
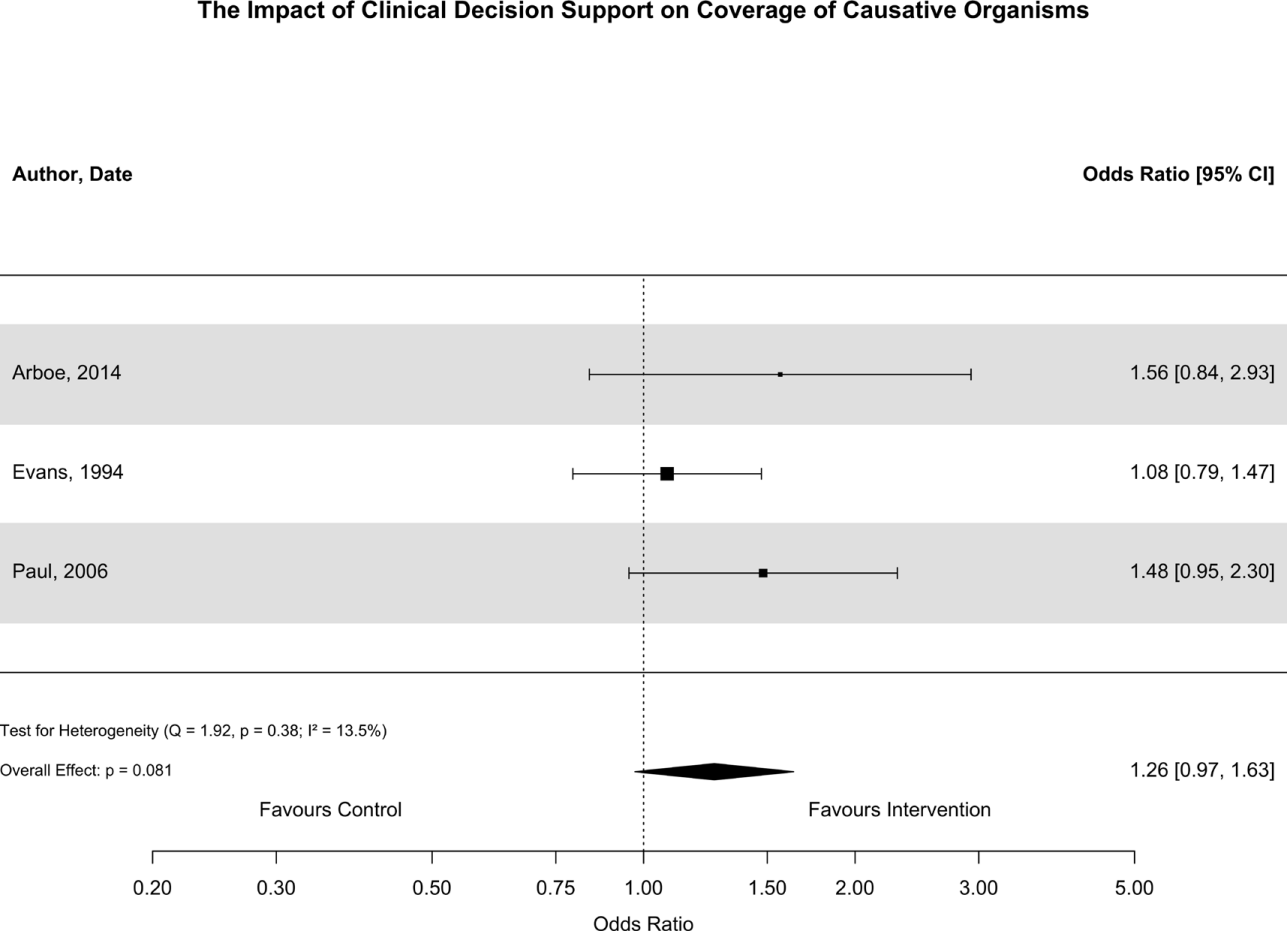
This forest plot summarises the outcomes of studies that reported the impact of CDSS implementation on coverage of the causative organism. Results from adjusted analyses were used to derive OR and 95% CIs in one study. Unadjusted ORs were derived from raw data in two studies. Appropriate coverage was similar before and after the implementation of CDSS (OR 1.26, 95% CI 0.97 to 1.63, p=0.081). There was minor statistical heterogeneity (I^2^=13.5%; Q=1.92, p=0.38). CDSS, Clinical Decision Support Software.

Guideline adherent antibiotic prescribing improved following the implementation of CDSS (OR 1.75, 95% CI 1.26 to 2.43, p<0.001). There was substantial heterogeneity associated with this result (I^2^=59.9%, p=0.07). This heterogeneity may have been related to the infectious syndrome the CDSS was used for. The results of three studies that explored the impact of CDSS implementation in pneumonia or skin and soft tissue infection identified a consistent improvement in guideline adherence of similar magnitude. However, the results of the one study that explored the use of CDSS for antibiotic prescribing in UTI did not identify any improvements in guideline adherent prescribing. There are also several other sources of heterogeneity including differences in study design, baseline guideline adherence, CDSS content and setting that may have also accounted for some of the differences observed. All studies were non-randomised; three were at serious risk of bias^23 24 28^ and one was at moderate risk of bias.^27^

Appropriate coverage of the causative organism was similar before and after the implementation of CDSS (OR 1.26, 95% CI 0.97 to 1.63, p=0.08). There was a small amount of heterogeneity associated with this result (I^2^=13.5%, p=0.38). Two of these studies were non-randomised and at serious risk of bias,^22 29^ and one was a cluster RCT at high risk of bias.^34^ Outcomes are summarised for individual studies in online supplemental material 6. Publication bias was not assessed due to the small number of studies in each subgroup.

### Antimicrobial stewardship

Outcomes relevant to antimicrobial stewardship were reported in six studies.^21 25 27 30 31 33^ Statistical analysis and reporting were too heterogeneous to allow for meta-analysis. Outcomes were related to total antibiotic use,^21^ length of antibiotic use,^25^ the use of specific antibiotics or groups of antibiotics where overuse is likely to contribute to AMR^21 27 30 31 33^ and the frequency of AMR organisms and *C. difficile.*^33^ It was consistently reported that the implementation of CDSS was associated with a reduction in the prescribing of broad-spectrum antibiotics.^21 27 30 31^ The results of the one other study that reported this outcome were also generally supportive of this association, but interpretation is more complex as the authors report segmented regression analysis with trend and level changes after three intervention phases.^33^ Notably, two of these studies were large cluster RCTs with approximately 100, 000 patients in each and were at low risk of bias.^30 31^ These two studies reported a decrease of empiric extended-spectrum antibiotic use of approximately 28% in patients with pneumonia and 17% in patients with UTI. One non-randomised study was at moderate risk of bias and reported a reduction in empiric extended-spectrum antibiotic use of 12%; however, this finding was not statistically significant (OR 0.88, 95% CI 0.75 to 1.04). One non-randomised study at serious risk of bias reported a significant reduction in total antibiotic use, carbapenem use and cephalosporin use in periods with CDSS, but no change in penicillin usage.^21^ Another non-randomised study reported outcomes related to the frequency of AMR organisms and *C. difficile* in addition to broad-spectrum antibiotic use.^33^ This study reported a decrease in the prescribing of piperacillin-tazobactam, carbapenems and other broad-spectrum antibiotics and a decrease in the incidence of *C. difficile* after the implementation of CDSS. However, this study was at serious risk of bias. A summary of results related to antimicrobial stewardship is provided in online supplemental material 7.

### Health economics

Health economic outcomes were reported in five studies. Four of these studies were non-randomised^22 23 25 29^ and at serious risk of bias. One study was a cluster RCT at high risk of bias.^34^ Several outcomes were reported, and many studies reported multiple outcomes. Outcomes included average or total costs of antibiotics,^23 25 29 34^ direct costs,^22 34^ costs of side effects,^34^ ecological costs^22 34^ and costs related to facility utilisation, pharmacy and laboratory costs.^25^ Health economic outcomes were extracted to reflect total costs if available, or antibiotic costs if total costs were unavailable. While not all studies provided statistical comparisons, there was no evidence that the implementation of CDSS was associated with cost savings. A summary of health economic outcomes is provided in online supplemental material 8.

## Discussion

This systematic review identified 15 studies that evaluated the impact of CDSS on empirical antibiotic prescribing and related outcomes. There was significant heterogeneity in study design, statistical analysis, intervention characteristics and outcomes reported. This heterogeneity precluded quantitative synthesis for most outcomes in this systematic review. Despite this, the evidence summarised in this review demonstrates that CDSS can be used to positively influence antibiotic prescribing in hospital.

### Clinical outcomes

This systematic review did not identify evidence of improvement in readmission rates or length of hospital stay following CDSS implementation. However, there was a trend towards reduced mortality following the implementation of CDSS (OR 0.76, 95% CI 0.57 to 1.01). A 24% relative reduction in mortality is substantial and necessitates further discussion, particularly as many acute infections treated in hospital have a significant absolute mortality rate.

In this context, CDSS are being evaluated as a therapeutic intervention, and the causal mechanisms through which the implementation of CDSS could result in a reduction in mortality need to be considered. Mortality secondary to acute infection is caused by the interaction of multiple factors. These include patient demographics, comorbidities, disease severity, the causative organism and antibiotic treatment. Only one of these factors is modifiable through the implementation of CDSS: antibiotic treatment. The study at lowest risk of bias which reported mortality outcome data did provide evidence that antibiotic treatment was modified following implementation of CDSS.^27^ There was a 6.7% increase in guideline-concordant antibiotic prescribing, mostly through a reduction in the prescribing of unnecessary broad-spectrum antibiotics, and an 8.5 min decrease in time from emeregency department (ED) admission to first antibiotics. While broad-spectrum antibiotic use is associated with worse outcomes,^52^ the magnitude of change in antibiotic prescribing is unlikely to account for a reduction in mortality of 38%, as reported in this study. In addition, in this study, there are significant baseline differences before and after deployment, with the post-deployment cohort being younger with fewer comorbidities and less severe disease. While the authors adjust for disease severity which incorporates many of these factors, it remains possible that these baseline differences account for some, or all, the observed differences in mortality.

Non-randomised study designs, which are common for the evaluation of CDSS, have inherent limitations that may account for differences that are not caused by implementation of CDSS. For example, mortality from acute infections, in particular respiratory infections, varies seasonally and temporally over longer periods. The mortality from community-acquired pneumonia in-hospital in the UK fell from 20.2% in 2009 to 10.4% in 2019. Irrespective of the reason for this mortality decrease, it is important that such temporal trends are accounted for in statistical analysis. Most studies reported in this systematic review do not adjust for most important baseline confounding variables or temporal trends, reducing the ability to assign causality to the implementation of the CDSS. Further high-quality studies are needed to prove that CDSS can improve clinical outcomes.

### Antibiotic appropriateness

This systematic review demonstrated that the implementation of CDSS was associated with improvements in guideline adherent antibiotic prescribing (OR 1.75, 95% CI 1.26 to 2.43) but similar coverage of causative organisms (OR 1.26, 95% CI 0.97 to 1.63). CDSS is most used as a tool to communicate information to clinical decision makers at the time it is needed. These results support that CDSS is an effective means to achieve this. While these studies are also at serious risk of bias, predominantly due to confounding, the extent to which this impacts the observed results is likely less than that for clinical outcomes as the relationship between intervention and outcome is more direct.

### Antimicrobial stewardship

Despite heterogeneity that precluded quantitative synthesis for these outcomes, there was a consistent reduction in prescribing of extended-spectrum antibiotics following the implementation of CDSS. Notably, two of these studies were at low risk of bias. These studies by Gohil and colleagues^30 31^ were cluster RCTs that randomised over 50 hospitals to the implementation of CDSS for antimicrobial stewardship. The studies were conducted concurrently over the same period and in the same hospitals. The CDSS provided an antimicrobial stewardship prompt for patients at low risk of infection secondary to a multi-drug-resistant organism (MDRO) who were about to be prescribed an extended-spectrum antibiotic. The risk of MDRO was calculated using a classification and regression tree algorithm using retrospective data separately for pneumonia^30^ and UTI,^31^ and different individual and organisational factors were associated with MDROs in each condition. The introduction of these prompts was associated with a relative reduction in broad-spectrum antibiotic prescribing of 28% in pneumonia and 17% in UTI. The baseline prescribing rate of broad-spectrum antibiotics was greater in the pneumonia cohort, which could partly account for the discrepancy in the effectiveness of these prompts, although there are also other plausible explanations. These prompts provided a simple and clear message integrated into the EHR and at the time of prescribing, likely contributing to their effectiveness. These studies provide the highest quality evidence that CDSS are effective in changing the prescribing behaviour of clinicians.

### Health economics

Health economic data were presented in five studies, but none of these were rigorous cost-effectiveness analyses. None of the studies that reported health economic outcomes identified significant changes in the cost of antibiotics or direct costs of care following the implementation of CDSS. Further high-quality cost-effectiveness analyses accompanying clinical outcomes are imperative if CDSS are to be recommended for widespread use.

### Limitations

There are methodological limitations to this systematic review. First, this systematic review focused on a specific aspect of antibiotic prescribing, initial empirical prescribing. While this was selected deliberately as an important aspect of antibiotic prescribing, it led to the exclusion of some CDSS that had a broader impact on antibiotic prescribing. Second, there was a relatively small number of studies that reported outcomes for each outcome group. Heterogeneity was observed in the outcomes of included studies across most outcome domains. There were several potential sources of heterogeneity including study design, setting, organisational context, time of implementation, statistical analysis, characteristics of the intervention and infectious syndrome. The small number of studies made it difficult to fully understand the cause of this heterogeneity and whether certain characteristics of CDSS were associated with effectiveness. Substantial heterogeneity has been observed in prior systematic reviews and meta-analyses exploring the effectiveness of CDSS more broadly^53^ and it is uncertain how CDSS can be designed with the best chance of success. Finally, the risk of bias of included studies was generally high and some of the observed results may be due to bias rather than the implementation of CDSS.

## Conclusion

The implementation of CDSS to guide empirical antibiotic prescribing in hospital can positively influence the prescribing of antibiotics, improve guideline adherence and reduce the prescribing of broad-spectrum antibiotics. Despite this, it is uncertain whether these changes translate to clinical benefit, predominantly because of limitations in study design resulting in high risks of bias. Further work is required to understand the variation in outcomes that result from CDSS implementation, to ensure that future systems are optimised.

## Supplementary material

10.1136/bmjopen-2025-099100 online supplemental file 1

## Data Availability

All data relevant to the study are available in the article, supplementary material, or cited references.
