# Supplementary material for: Impact of clinical decision support software on empirical antibiotic prescribing and patient outcomes: a systematic review and meta-analysis
Source: BMJ Open. 2025 Nov 27;15(11):e099100. doi: 10.1136/bmjopen-2025-099100 (PMC12666123; doi:10.1136/bmjopen-2025-099100)
Supplement: online supplemental file 1 [file bmjopen-15-11-s001.docx]

**Supplementary Material Contents**

**Supplementary 1 – Literature Search Strategies -** *page 2-5*

**Supplementary 2 – Description of Clinical Decision Support Systems** *- page 6-12*

**Supplementary 3 - Description of Individual Studies and Risk of Bias for Individual Outcomes** *-page 13-37*

**Supplementary 4 – Mortality Meta-analysis Funnel Plot -** *page 43*

**Supplementary 5 – Summary of Clinical Outcomes** *- page 44-48*

**Supplementary 6 – Summary of Antibiotic Appropriateness Outcomes** – *page 49-50*

**Supplementary 7 – Summary of Antimicrobial Stewardship Outcomes** – *page 51-55*

**Supplementary 8 – Summary of Health Economic Outcomes –** *page 56*

**Supplementary 9 – PRISMA Checklist** – *page 57-60*

**Supplementary 1 – Literature search strategies**

**Ovid MEDLINE Search Terms**

Ovid MEDLINE(R) <1946 to January Week 4 2024>

1 exp Drug Therapy, Computer-Assisted/ 1693

2 exp Decision Making, Computer-Assisted/ 134434

3 exp Medical Order Entry Systems/ 2486

4 exp Electronic Prescribing/ 1196

5 (exp artificial intelligence/ or exp machine learning/) and (decision support.mp. or exp Decision Support Systems, Clinical/) [mp=title, book title, abstract, original title, name of substance word, subject heading word, floating sub-heading word, keyword heading word, organism supplementary concept word, protocol supplementary concept word, rare disease supplementary concept word, unique identifier, synonyms, population supplementary concept word, anatomy supplementary concept word] 5343

6 exp Medication Errors/ and (electronic or digital$ or computer$ or automat$ or "artificial intelligence" or "machine learning").mp. [mp=title, book title, abstract, original title, name of substance word, subject heading word, floating sub-heading word, keyword heading word, organism supplementary concept word, protocol supplementary concept word, rare disease supplementary concept word, unique identifier, synonyms, population supplementary concept word, anatomy supplementary concept word] 3651

7 ((Electronic or digital or computer$ or medic$ or prescri$ or automat$) adj4 (alert$ or prompt$ or recommendation$ or reminder$ or feedback or notif$ or expert system$)).mp. [mp=title, book title, abstract, original title, name of substance word, subject heading word, floating sub-heading word, keyword heading word, organism supplementary concept word, protocol supplementary concept word, rare disease supplementary concept word, unique identifier, synonyms, population supplementary concept word, anatomy supplementary concept word] 20805

8 ((electronic or digital$ or computer$ or automat$) adj3 guideline$).mp. [mp=title, book title, abstract, original title, name of substance word, subject heading word, floating sub-heading word, keyword heading word, organism supplementary concept word, protocol supplementary concept word, rare disease supplementary concept word, unique identifier, synonyms, population supplementary concept word, anatomy supplementary concept word] 1486

9 (computer$ adj2 ("order entry" or order-entry or CPOE)).mp. [mp=title, book title, abstract, original title, name of substance word, subject heading word, floating sub-heading word, keyword heading word, organism supplementary concept word, protocol supplementary concept word, rare disease supplementary concept word, unique identifier, synonyms, population supplementary concept word, anatomy supplementary concept word] 1711

10 (CDST or CDSS).mp. [mp=title, book title, abstract, original title, name of substance word, subject heading word, floating sub-heading word, keyword heading word, organism supplementary concept word, protocol supplementary concept word, rare disease supplementary concept word, unique identifier, synonyms, population supplementary concept word, anatomy supplementary concept word] 2109

11 ((clinical or computer$ or electronic or digital or automat$ or artificial intelligence or machine learning) adj7 decision support).mp. [mp=title, book title, abstract, original title, name of substance word, subject heading word, floating sub-heading word, keyword heading word, organism supplementary concept word, protocol supplementary concept word, rare disease supplementary concept word, unique identifier, synonyms, population supplementary concept word, anatomy supplementary concept word] 17428

12 exp Decision Support Systems, Clinical/ 9655

13 exp Expert Systems/ 3506

14 1 or 2 or 3 or 4 or 5 or 6 or 7 or 8 or 9 or 10 or 11 or 12 or 13 178223

15 exp Anti-Bacterial Agents/ 828798

16 (antibiotic$ or antimicrob$).mp. [mp=title, book title, abstract, original title, name of substance word, subject heading word, floating sub-heading word, keyword heading word, organism supplementary concept word, protocol supplementary concept word, rare disease supplementary concept word, unique identifier, synonyms, population supplementary concept word, anatomy supplementary concept word] 549009

17 15 or 16 1069263

18 14 and 17 2567

**Embase Search Terms**

Embase <1974 to 2024 February 02>

1 exp computer assisted drug therapy/ 942

2 exp decision support system/ 36020

3 exp clinical decision support system/ 6559

4 exp computerized provider order entry/ 6652

5 exp electronic prescribing/ 4495

6 exp expert system/ 5890

7 (exp artificial intelligence/ or exp machine learning/) and (decision support.mp. or exp decision support system/ or exp clinical decision support systems/) 9207

8 exp Medication Errors/ and (electronic or digital$ or computer$ or automat$ or "artificial intelligence" or "machine learning").mp. [mp=title, abstract, heading word, drug trade name, original title, device manufacturer, drug manufacturer, device trade name, keyword heading word, floating subheading word, candidate term word] 4971

9 ((Electronic or digital or computer$ or medic$ or prescri$ or automat$) adj4 (alert$ or prompt$ or recommendation$ or reminder$ or feedback or notif$ or expert system$)).mp. [mp=title, abstract, heading word, drug trade name, original title, device manufacturer, drug manufacturer, device trade name, keyword heading word, floating subheading word, candidate term word] 39016

10 ((electronic or digital$ or computer$ or automat$) adj3 guideline$).mp. [mp=title, abstract, heading word, drug trade name, original title, device manufacturer, drug manufacturer, device trade name, keyword heading word, floating subheading word, candidate term word] 2420

11 ((computer$ adj2 (order entry or order-entry)) or CPOE).mp. [mp=title, abstract, heading word, drug trade name, original title, device manufacturer, drug manufacturer, device trade name, keyword heading word, floating subheading word, candidate term word] 4145

12 (CDST or CDSS).mp. [mp=title, abstract, heading word, drug trade name, original title, device manufacturer, drug manufacturer, device trade name, keyword heading word, floating subheading word, candidate term word] 3504

13 ((clinical or computer$ or electronic or digital or automat$ or artificial intelligence or machine learning) adj7 decision support).mp. [mp=title, abstract, heading word, drug trade name, original title, device manufacturer, drug manufacturer, device trade name, keyword heading word, floating subheading word, candidate term word] 22786

14 1 or 2 or 3 or 4 or 5 or 6 or 7 or 8 or 9 or 10 or 11 or 12 or 13 96913

15 (antibiotic$ or antimicrob$).mp. [mp=title, abstract, heading word, drug trade name, original title, device manufacturer, drug manufacturer, device trade name, keyword heading word, floating subheading word, candidate term word] 1133728

16 exp antibiotic agent/ 1850671

17 15 or 16 2193750

18 14 and 17 6695

**Cochrane Central Register of Controlled Trials (CENTRAL) Search Terms**

Search Name: CDSS Antibiotics Cochrane

Last Saved: 05/02/2024 11:51:02

ID Search

#1 MeSH descriptor: [Drug Therapy, Computer-Assisted] explode all trees

#2 MeSH descriptor: [Decision Making, Computer-Assisted] explode all trees

#3 MeSH descriptor: [Decision Support Systems, Clinical] explode all trees

#4 MeSH descriptor: [Medical Order Entry Systems] explode all trees

#5 MeSH descriptor: [Electronic Prescribing] explode all trees

#6 MeSH descriptor: [Expert Systems] explode all trees

#7 MeSH descriptor: [Artificial Intelligence] explode all trees

#8 MeSH descriptor: [Machine Learning] explode all trees

#9 #7 or #8

#10 MeSH descriptor: [Decision Support Systems, Clinical] explode all trees

#11 ("decision support"):ti,ab,kw

#12 #10 or #11

#13 #9 and #12

#14 MeSH descriptor: [Medication Errors] explode all trees

#15 (electronic or digital* or computer* or automat* or "artificial intelligence" or "machine learning"):ti,ab,kw

#16 #14 and #15

#17 ((Electronic or digital or computer* or medic* or prescri* or automat*) NEAR/4 (alert* or prompt* or recommendation* or reminder* or feedback or notif* or expert system*)):ti,ab,kw

#18 ((electronic or digital* or computer* or automat*) NEAR/3 (guideline*)):ti,ab,kw

#19 (((computer* NEAR/2 (order entry or order-entry)) or CPOE)):ti,ab,kw

#20 (CDST or CDSS):ti,ab,kw

#21 ((clinical or computer* or electronic or digital or automat* or “artificial intelligence” or “machine learning”) NEAR/7 (“decision support”)):ti,ab,kw

#22 #1 or #2 or #3 or #4 or #5 or #6 or #13 or #16 or #17 or #18 or #19 or #20 or #21

#23 MeSH descriptor: [Anti-Bacterial Agents] explode all trees

#24 (antibiotic* or antimicrob*):ti,ab,kw

#25 #23 or #24

#26 #22 and #25

**Supplementary 2 – Description of Clinical Decision Support Systems**

| Reference & CDSS name* | Intervention Description | CDSS Class | Integration within EHR | Individual Patient Data Utilised | Stakeholder involvement in Design | Pilot Testing | Concurrent Interventions |
| --- | --- | --- | --- | --- | --- | --- | --- |
| Al Bahar, 2020 | Structured prescribing that provides recommendations regarding antibiotic choice, dose, duration, and route of administration for empirical treatment derived from hospital guidelines. | Knowledge-based | Integrated into the EHR and prescribing system | Includes weight, renal function, hepatic function, allergies, microbiology susceptibility results. | System developed and validated by group of Infectious Diseases Physicians, Microbiologists, Infection Control Specialists, Pharmacists, and Informatics team members. | None specified. | Frequent informal teaching seminars for senior and junior prescribers delivered by pharmacists and informatics specialists. |
| Arboe, 2014  *TREAT* | TREAT is a CDSS that uses a causal probabilistic network to provide recommendations for empirical antibiotic prescribing. It also presents the probability of infection, its source, pathogen distributions, and mortality probabilities. | Non-knowledge based | TREAT extracts patient data but it is unclear whether it is integrated into the electronic health record and prescribing system. | Automatically imported data – age, gender, leucocyte count, neutrophil count, haemoglobin, platelets, C-reactive protein, sodium, potassium, microbiology findings.  Manually entered – background, history of illness, recent admissions, signs and symptoms, radiology findings | Not clearly specified. | Not explicitly reported. | Group education of clinicians & one-on-one help available. |
| Buising, 2008 | The CDSS guided site of care decisions, and provided antibiotic advice including empirical antibiotic choice, duration, timing of oral to IV switch. | Knowledge - based | Accessed via hospital computers and linked to hospital databases to extract patient information but not integrated in EHR. | Pneumonia Severity Index, CURB-65, allergy status. It is unclear what specifically was used to generate antibiotic advice. | Not specified. | Not specified. | Academic detailing preceded CDSS intervention and some activities continued. |
| Carman, 2011 | Manually entered patient data used to recommend whether empiric antibiotic coverage for MRSA is appropriate or not. | Knowledge-based | Integrated into EHR and prescribing system | History and examination findings entered manually including presence of cellulitis, size of abscess, systemic illness, risk factors for recurrent MRSA. | Not reported. | Providers encouraged to provide feedback and recommendation for modification. Some minor revision made. | The CDSS also has functionality to recommended investigation (wound culture) and prescription of chlorhexidine.  Project information was disseminated via email and via presentation.  New EMR implemented directly before intervention. |
| Ciarkowski, 2020 | CDSS initiated by order of antibiotic and chest x-ray and provides guidance on site of care, tools for assessing risk of drug resistant pathogens, investigations, and recommended empiric antibiotics | Knowledge-based | Integrated into the order entry system and initiated when an order for an antibiotic and chest x-ray is made | This is not clearly specified but individual risk of drug-resistant pathogens and site of care were likely used. | Developed by multidisciplinary team including physicians, pharmacists, value engineering, and information technology. | The CDSS was initially validated by a single ED provider during a 3-month pilot test before full deployment. | CDSS was tested in two phases. One phase included audit and feedback and antimicrobial stewardship services. In another phase stewardship and audit and feedback were scaled back. |
| Dean, 2015  *ePNA* | ePNA is a CDSS that detects the presence of likely pneumonia and recommends site of care (outpatient, hospital, ICU) and empirical antibiotics (agent, dose, duration, route) and investigations | Knowledge-based (for antibiotic recommendation aspect of CDSS) | Integrated into the EHR and automatically pulled real-time clinical data from the EHR. | Pneumonia severity scores, PaO2:FiO2 ratio, recent hospitalisation, admission source, previous microbiology results, CXR report | Emergency physicians and nurses involved in development and deployment. There was collaboration with ED leadership team. | The tool underwent pilot testing in one hospital before full deployment. Feedback from pilot phase led to refinements to improve usability. | One on one academic detailing to receive feedback and raise awareness of tool. Study authors were available to answer questions about the tool. |
| Dean, 2022  *ePNA* | ePNA is a CDSS that recommends site of care (outpatient, hospital, ICU) and empirical antibiotics (agent, dose, duration, route) and investigations | Knowledge-based | Alert sent to clinician in EHR if probability of pneumonia reaches certain threshold based on patient data. Order set to prescribe antibiotics can be accessed from CDSS. | Drug resistance in pneumonia (DRIP) score, site of care. | Not specified here. Previous publication stated that ED leadership team involved in development and deployment. | Feedback sought using physician champions and nurse educators. Beta testing performed July 2017. | Frontline provider education, audit and feedback, email reminders. |
| Demonchy, 2014 | The CDSS provided recommendations regarding investigations, indications for hospitalisation, antibiotic treatment, and follow up. | Knowledge-based | The CDSS was integrated into the EHR and was automatically triggered when a UTI diagnosis was validated. | Unclear what individual patient data was used. Authors state that recommendations tailored to individual patient data. | Designed by two infectious diseases specialists and a public health and health informatics specialist. | Pilot testing was performed by one project leader in each ED. Potential barriers to use were identified and addressed. | Introductory demonstration provided to ED staff at grand round. Leaders in each ED provided informal demonstrations. |
| Evans, 1994 | CDSS uses patient information and past microbiology results to determine the most likely pathogen and displays 5 treatment regimens likely to be effective for all pathogens. | Methods not clearly reported. | The CDSS extracts patient information from the computer based patient record. Unclear if the CDSS is integrated in the EHR. | Admission diagnosis, white-cell count, renal dysfunction, temperature, surgical data, chest radiograph, and information from pathology, serology, microbiology reports, allergies | Not described in this paper. | Questionnaire data reported in this study and reported as ‘uniformly positive’. | None specified. |
| Gohil, 2024a  *INSPIRE* | The CDSS displays an antimicrobial stewardship prompt when extended spectrum antibiotics are requested for patients with pneumonia and their individual risk of multi-drug-resistant organisms is below 10%. The prompt advises that the extended spectrum antibiotic is not recommended and suggests a recommended antibiotic. | Non-knowledge based (Classification and Regression Tree algorithm used to establish individual risk of MDRO) | Integrated into the EHR/ e-prescribing system. | Authors state that 50+ variables were used to develop the models. Risk factors of absolute risk of MDRO over 10% include patient history of MRSA and pseudomonas. | Authors state that the CDC and HCA had a role in the design and conduct of the study | Not reported. | Education provided to both groups about national standards and updated guidance during trial period. Both groups coached to avoid competing interventions. The intervention group received education on how MDRO risk estimates were calculated. Feedback reports on extended spectrum antibiotics provided. |
| Gohil, 2024b  *INSPIRE* | The CDSS displays an antimicrobial stewardship prompt when extended spectrum antibiotics are requested for patients with UTI and their individual risk of multi-drug-resistant organisms is below 10%. The prompt advises that the extended spectrum antibiotic is not recommended and suggests a recommended antibiotic. | Non-knowledge based (Classification and Regression Tree algorithm used to establish individual risk of MDRO) | Integrated into the EHR/ e-prescribing system. | Multiple patient and facility risk factors input into CART model. Risk factors that predicted 10%+ risk of MDRO in UTI included history of MRSA/ VRE/ pseudomonas/ ESBL, facility MRSA/ VRE rate, sex, age, Medicaid. | Authors state that the CDC and HCA had a role in the design and conduct of the study | Not reported. | Education provided to both groups about national standards and updated guidance during trial period. Both groups coached to avoid competing interventions. The intervention group received education on how MDRO risk estimates were calculated. Feedback reports on extended spectrum antibiotics provided. |
| Leibovici, 2013  *TREAT* | TREAT is a CDSS that uses a causal probabilistic network to provide recommendations for empirical antibiotic prescribing. It also presents the probability of infection, its source, pathogen distributions, and mortality probabilities. | Non-knowledge based | Linked to the EHR as data is extracted but no clear evidence that it is integrated into the EHR or electronic prescribing system. | Patient demographics, comorbidities, devices (e.g. presence of catheter), vital signs, laboratory tests, symptoms and signs relevant to infection, available radiological (e.g. chest X-ray) and microbiological (e.g. Gram stain) results | Not clearly specified. | A prospective cohort study is described prior to the RCT. | The CDSS was presented to prescribers at time of prescribing. |
| Ng, 2022 | The CDSS guides empirical antibiotic choice for 52 infectious syndromes. | Knowledge-based | The CDSS is accessed via the electronic medication record. Prescribers specify the infection and selected patient parameters to received a recommendation based on empirical prescribing guidelines. | Data used not specified. Authors state selected patient parameters and the indication are used. | Discussed in separate paper – there was input from clinical departments. | Discussed in separate paper – data was collected about user acceptability. | Prescription review and feedback |
| Paul, 2006  *TREAT* | TREAT is a CDSS that uses a causal probabilistic network to provide recommendations for empirical antibiotic prescribing. It also presents the probability of infection, its source, pathogen distributions, and mortality probabilities. | Non-knowledge based | Linked to the EHR as data is extracted but no clear evidence that it is integrated into the EHR or electronic prescribing system. | Patient demographics, comorbidities, devices (e.g. presence of catheter), vital signs, laboratory tests, symptoms and signs relevant to infection, available radiological (e.g. chest X-ray) and microbiological (e.g. Gram stain) results | Not clearly specified. | A prospective cohort study is described prior to the RCT. | The CDSS was presented to prescribers at time of prescribing. |
| Rohrig, 2008 | Guide empirical antibiotic prescribing based on hospital guidelines. | Knowledge-based | Integrated module within the electronic patient record | Description suggests individual patient data used but it is not clear what patient data used. | Limited description in previous paper stated cooperation with Medical Microbiology and Virology | Previous usability testing reported separately. | None specified. |

*Supplementary 2 describes key elements of the CDSS included in this study including their functionality, integration, and design. EHR: Electronic Health Record; CDSS: Clinical Decision Support Software; IV: Intravenous; MRSA: Methicillin-resistant Staphylococcus Aureus; EMR: Electronic Medical Record; ICU: Intensive Care Unit; PaO2: Partial pressure of oxygen; FiO2: Fraction of inspired oxygen; CXR: Chest X-ray; ED: Emergency Department; UTI: Urinary Tract Infection; MDRO: Multi-Drug Resistant Organism; CDC: Centers for Disease Control and Prevention; CART: Classification and Regression Tree; VRE: Vancomycin-Resistant Enterococci; ESBL: Extended-Spectrum Beta-Lactamase. CURB-65 is a scoring system that is used to stratify pneumonia severity. ePNA refers to the name of a pneumonia CDSS in the respective studies. TREAT refers to the name of a CDSS in the respective studies.*

*CDSS name provided where it has been named and used across more than one study to aide with comparability.

**Supplementary 3 – Description of Individual Studies and Risk of Bias for Individual Outcomes**

| Al Bahar, 2020 | | |
| --- | --- | --- |
| Study design | Uncontrolled before-after study (retrospective). | |
| Participants | No specific infectious syndrome. Analysis includes all antibiotic prescriptions in hospital. | |
| Intervention | Structured prescribing in the electronic health record/ prescribing system that uses individual patient data to provide guideline adherent empirical antibiotic prescribing advice. | |
| Outcome(s) extracted | O1 - Total antibiotic usage  O2 - Penicillin usage  O3 - Cephalosporin usage  O4 - Carbapenem usage | |
| *Risk of Bias (ROBINS-I)* | | |
| Bias Domain | **Authors’ judgement** | **Supporting information** |
| *Bias due to confounding* | O1 – O4 - Serious | O1 – O4 - Retrospective uncontrolled before-after study with no adjustment for confounders. |
| *Bias due to selection of participants* | O1 – O4 -Moderate | O1 – O4 - The follow up of participants does not begin when the intervention was introduced. It is not clearly specified why this is the case. |
| *Bias in classification of interventions* | O1 – O4 - Low | O1 – O4 - There was a clear period of availability of structured prescribing which is well recorded and therefore the distinction between intervention and control is clear. |
| *Bias due to deviation from interventions* | O1 – O4 -Low | O1 – O4 - There is limited reporting about the possibility of deviations from the intended intervention. Given the nature of the intervention, it is likely any deviations were in keeping with what would be expected in clinical practice. |
| *Bias due to missing data* | O1 – O4 -Moderate | O1 – O4 - Outcome data was extracted from the electronic patient database and is likely to be relatively complete. However, there is limited reporting to confirm this. |
| *Bias in measurements of outcomes* | O1 – O4 - Low | O1 – O4 - It is not specified whether outcome assessors were blinded although the outcome measures are objective and are unlikely to be prone to bias. |
| *Bias in selection of reported result* | O1 – O4 -Moderate | O1 – O4 - There is no a priori statistical analysis plan and results presented include multiple different antibiotic subgroups. These do represent broad classes of antibiotics likely to be relevant to antimicrobial stewardship, but there is limited discussion about how these were selected. |
| *Overall risk of bias* | O1 – O4 - Serious |  |
| *Other comments* | Analysis included all antibiotic prescriptions in hospital and therefore included antibiotics prescribed in outpatient clinic settings and prophylactic antibiotics. However, this study was still included as the primary aim of the intervention was to guide empirical antibiotic prescribing, even though the outcome did not capture only this effect.  Statistical analysis is suboptimal to answer this research question. | |

| Arboe, 2014 | | |
| --- | --- | --- |
| Study design | Uncontrolled before-after study (separate retrospective and prospective studies) | |
| Participants | Patients with acute infection on an acute medical ward (53% pneumonia, 18% UTI, 8% skin/ soft-tissue, 4% other) | |
| Intervention | TREAT – this intervention is based on a causal probabilistic network that provides the probability of infection, severity, source of infection, pathogen distribution, mortality, and antibiotic coverage. TREAT recommends the antibiotics with the highest cost-benefit. | |
| Outcome(s) extracted | O1 – 30-day mortality  O2 – length of hospital stay  O3 – appropriateness of antibiotic  O4 – antibiotic cost | |
| *Risk of Bias (ROBINS-I)* | | |
| Bias Domain | **Authors’ judgement** | **Supporting information** |
| *Bias due to confounding* | O1 -O4 | O1 – O4 - Uncontrolled before-after study with very limited adjustment for confounding. |
| *Bias due to selection of participants* | O1 + O2 – Moderate  O3 – Serious  O4 - Serious | O1 – O2 - There is limited reporting about how patients were selected for inclusion in each part of the study and whether inclusion criteria were the same for both parts of the study. Whilst there is no clear use of post baseline variables, reporting is limited, and it is plausible that selection bias exists.  O3 – O4 – Only patients with positive microbiology results are included in results. Inclusion criteria is not explicit and culture positivity and ordering could be influenced by the introduction of the intervention. |
| *Bias in classification of interventions* | O1 -O4 - Low | O1 – O4 - There is a clear time point when the intervention was introduced, and classification is likely to be accurate. |
| *Bias due to deviation from interventions* | O1 – O4 - Low | O1 – O4 – There is no evidence of deviation from the intervention outside that expected in clinical practice. |
| *Bias due to missing data* | O1 – O4 - Low | O1 – O4 – No missing data is explicitly reported and all participants appear to be accounted for in analysis. |
| *Bias in measurements of outcomes* | O1 + O2 – Low  O3 – Serious  O4 - Low | O1, O2, O4 – Objective outcomes that are unlikely to be biased through non blinding of outcome assessors.  O3 - It is unclear whether the outcome assessors were blind to the intervention. Given there is subjectivity in determining whether treatment was appropriate this could introduce bias. |
| *Bias in selection of reported result* | O1 – O4 – Serious | O1 – O4 - There is no a priori statistical analysis plan available. There is evidence of analysis including multiple different measures of outcomes and multiple subgroup analyses. |
| *Overall risk of bias* | O1 – O4 - Serious | |
| *Other comments* | Multiple outcomes are reported in this paper. However, only those that represent the intention to treat effect were extracted. Many outcomes compare what would have happened should the advice of TREAT been followed, which does not reflect real life practice. | |

*UTI: Urinary Tract Infection. TREAT refers to the name of the CDSS evaluated in this study.*

| Buising, 2008 | | |
| --- | --- | --- |
| Study design | Uncontrolled before-after study/ interrupted time series | |
| Participants | Patients presenting to the ED with pneumonia | |
| Intervention | The CDSS provided recommendations regarding antibiotic choice, site of care, and highlighted patients likely to need ICU review. | |
| Outcome(s) extracted | O1 – Time to antibiotics  O2 – Mortality  O3 – Length of stay  O4 – Appropriate antibiotic coverage  O5 – Antibiotic cost | |
| *Risk of Bias (ROBINS-I)* | | |
| Bias Domain | **Authors’ judgement** | **Supporting information** |
| *Bias due to confounding* | O1 – O5 - Serious | O1 – O5 – Uncontrolled before-after study/ ITS analysis. The authors adjust for potential confounding variably across different outcomes. Adjustment includes age, disease, severity, and suspected aspiration for primary analysis. This does not account for time trends and comorbidities, for example. |
| *Bias due to selection of participants* | O1 – O5 - Low | O1 – O5 - There is no evidence of the use of post baseline variables to select patients differentially in control and intervention groups. |
| *Bias in classification of interventions* | O1 – O5 - Low | O1 – O5 - The intervention period is clearly defined based on date of implementation. |
| *Bias due to deviation from interventions* | O1 – O5 - Low | O1 – O5 - There is no evidence of deviation from the intended interventions outside what would be expected in normal clinical practice. |
| *Bias due to missing data* | O1 – O5 - Moderate | O1 -O5 - There is evidence of missing data for some patient outcomes such as mortality, and for other outcomes the number of patients included in analysis is not explicitly reported. |
| *Bias in measurements of outcomes* | O1 – O5 - Low | O1 – O5 – objective outcomes were used and therefore bias from non-blinding of outcome assessors is unlikely. |
| *Bias in selection of reported result* | O1 – O5 - Serious | O1 – O5 - There is no a priori statistical analysis plan. There are also indications that suggest selective reporting is possible such as the reporting of multiple subgroup analyses and analysis of the primary outcome with different methods (logistic regression and ITS). |
| *Overall risk of bias* | O1 – O5 - Serious | |
| *Other comments* | Multiple subgroup analyses reported for the primary outcome (antibiotic appropriateness) based on severity of pneumonia. Only the primary outcome has been extracted as this was the most relevant to this systematic review, and other sub-group analyses are all in keeping with this result. Another outcome reported by authors includes the proportion of patients who went direct from ED to ICU. This was not felt to be relevant to this review question.  Unadjusted results were included in the mortality meta-analysis as the results from adjusted analyses only present a p value and raw values. Adjusted values were used for the guideline adherence meta-analysis. The results presented compared CDSS to academic detailing. Adjusted results were not presented for CDSS to baseline. | |

*ED: Emergency Department; ICU: Intensive Care Unit; CDSS: Clinical Decision Support Software; ITS: Interrupted Time Series.*

| Carman, 2011 | | |
| --- | --- | --- |
| Study design | Uncontrolled before-after study | |
| Participants | Patients with skin and soft tissue infection (abscess) presenting to ED | |
| Intervention | CDSS recommended whether empirical coverage for MRSA should be provided | |
| Outcome(s) extracted | Guideline adherent antibiotic prescribing | |
| *Risk of Bias (ROBINS-I)* | | |
| Bias Domain | **Authors’ judgement** | **Supporting information** |
| *Bias due to confounding* | Serious | Uncontrolled before-after study with adjustment only for age and sex. Between the baseline period of this study and the postintervention period a new EHR was introduced which could have also impacted on prescribing behaviour. |
| *Bias due to selection of participants* | Serious | Patients were included in the study if they presented with a chief complaint of abscess. It is unclear how this information was obtained. It is possible that differences in coding practices in the control and intervention period could cause a selection bias, particularly as new EHR system was introduced. |
| *Bias in classification of interventions* | Low | Intervention clearly defined by separate time periods before and after intervention. |
| *Bias due to deviation from interventions* | Low | No evidence of deviation from intervention beyond that expected in normal clinical practice. |
| *Bias due to missing data* | Serious | Evidence of missing data about patient sex that is only present in the intervention arm. This was used as a covariate in analysis and therefore could have biased results. |
| *Bias in measurements of outcomes* | Moderate | There is limited detail provided about outcome assessment, and it is unclear if outcome assessors were blinded. However, outcome is objective and unlikely to be biased due to non-blinding. |
| *Bias in selection of reported result* | Moderate | No a priori statistical analysis plan available. |
| *Overall risk of bias* | Serious | |
| *Other comments* | Other outcomes included in this study were chlorhexidine use, appropriateness of cultures, and user satisfaction of CDSS. These do not answer the question of the systematic review as they are unrelated to antibiotic prescribing and thus were not included.  Adjusted results were used for meta-analysis of guideline adherent antibiotic prescribing. P-value reported in text as 0.000. Conservative estimate of p = 0.0009 used for p-value. | |

*ED: Emergency Department; CDSS: Clinical Decision Support Software; MRSA: Methicillin-Resistant Staphylococcus Aureus; EHR: Electronic Health Record.*

| Ciarkowski, 2020 | | |
| --- | --- | --- |
| Study design | Uncontrolled before-after study (retrospective) | |
| Participants | Patients with pneumonia who attended ED and were admitted to the hospitalist/ pulmonology acute care or intensive care units | |
| Intervention | CDSS that provided guidance on appropriate triage, risk of drug-resistant pathogens, diagnostic testing, and recommended empiric therapy | |
| Outcome(s) extracted | O1 – inpatient mortality  O2 – 30-day readmission  O3 – length of hospital stay  O4 – length of IV antibiotics  O5 – total length of antibiotics  O6 – total cost per visit | |
| *Risk of Bias (ROBINS-I)* | | |
| Bias Domain | **Authors’ judgement** | **Supporting information** |
| *Bias due to confounding* | O1 – O6 - Serious | O1 – O6 - Whilst analysis does adjust for several important confounders including age, comorbidities, disease severity, and season, secular trends are an important confounder that are not adjusted for in analysis. |
| *Bias due to selection of participants* | O1 – O6 - Serious | O1 – O6 - Participants are included based on the site they receive care and the presence of a diagnosis of pneumonia. This CDSS influences the site of care and therefore may cause a selection bias. It may also indirectly change the rates of pneumonia diagnoses causing selection bias. |
| *Bias in classification of interventions* | O1 – O6 - Low | O1 – O6 - Intervention groups were clearly defined based on time intervention was implemented. This is likely to be accurately recorded. |
| *Bias due to deviation from interventions* | O1 – O6 -Low | O1 – O6 - No evidence of deviation from intended interventions. |
| *Bias due to missing data* | O1 – O6 - No Information | O1 – O6 - Outcome data was extracted from the electronic patient database and is likely to be relatively complete however this is not clearly reported. |
| *Bias in measurements of outcomes* | O1 – O6 - Low | O1 – O6 - It is unclear if outcome assessors were blinded but this is unlikely to bias results as outcomes are objective |
| *Bias in selection of reported result* | O1 – O6 - Moderate | O1 – O6 – There is no clear evidence of multiple analyses with biased selection of the reported result but there is no a priori statistical analysis plan. |
| *Overall risk of bias* | O1 – O6 - Serious | |
| *Other comments* | Other outcomes reported but not extracted include length of inpatient azithromycin therapy, length of inpatient atypical antibiotic therapy, procalcitonin lab utilization, and other cost outcomes. These were not included as they were either not directly related to empirical antibiotic prescribing (lab utilisation) or reflected a subgroup analysis of included outcomes.  For meta-analysis of mortality, the estimated marginal means reported in the study were used to derive an estimated adjusted Odds Ratio. This was used together with the reported p value for meta-analysis. | |

*ED: Emergency Department; CDSS: Clinical Decision Support Software; IV: Intravenous.*

| Dean, 2015 | | |
| --- | --- | --- |
| Study design | Controlled before-after study | |
| Participants | Patients with a diagnosis of pneumonia | |
| Intervention | ePNA – a CDSS that influences antibiotic choice, site of care, and investigations in pneumonia. | |
| Outcome(s) extracted | O1 – 30-day all-cause mortality  O2 – length of hospital stay  O2 – readmission to hospital  O4 – guideline adherent patient disposition | |
| *Risk of Bias (ROBINS-I)* | | |
| Bias Domain | **Authors’ judgement** | **Supporting information** |
| *Bias due to confounding* | O1 – Moderate  O2 – O4 – Serious | O1 - The authors address confounding using a (non-randomised) control group of EDs where the intervention was not implemented and adjustment for important confounders including disease severity, sex, and baseline differences in event rates and severity over time. However, it is still plausible that adjustment was incomplete and residual confounding exists.  O2 -O4 - Statistical analysis for other outcomes was reported less clearly and it does not appear that confounding was adjusted for completely. |
| *Bias due to selection of participants* | O1 – O4 - Moderate | O1 – O4 - The selection of participants was made based on the presence of an ICD-9 code of pneumonia and CXR findings. It is possible that introduction of the CDSS also changed the coding of pneumonia and introduced a selection bias. |
| *Bias in classification of interventions* | O1 – O4 - Low | O1 – O4 - Intervention status based on known time of implementation |
| *Bias due to deviation from interventions* | O1 – O4 - Low | O1 – O4 – There is no clear deviation from intended interventions. |
| *Bias due to missing data* | O1 – Moderate  O2 – O4 - Low | O1 - Over 10% of patients were missing blood urea level data, which is an important aspect of disease severity calculation. Urea values were imputed for these patients. There is limited reporting about whether this missing data was equally distributed across intervention and control arms and pre- and post-deployment and therefore is a source of bias.  O2 -O4 - The authors reported missing data for blood urea levels and vital signs, but these were not used in this analysis. No missing outcome data is reported. |
| *Bias in measurements of outcomes* | O1 – O4 - Low | O1 – O4 – Objective outcomes unlikely to be prone to outcome measurement bias. |
| *Bias in selection of reported result* | O1 – Serious  O2 – O4 - Moderate | O1 – O4 - There is no a priori statistical analysis plan for any outcome and post hoc analysis was performed using different statistical methods and in different subgroups for 30-day mortality |
| *Overall risk of bias* | O1 – O4 - Serious | |
| *Other comments* | Inpatient mortality is reported in addition to 30-day mortality. Only 30-day mortality was extracted as this represents primary outcome and results are adjusted for important confounders. | |

*ED: Emergency Department; CDSS: Clinical Decision Support Software; CXR: Chest X-ray; ICD-9: International Classification of Diseases Ninth Edition.*

| Dean, 2022 | | |
| --- | --- | --- |
| Study design | Non-randomised stepped-wedge cluster-controlled clinical trial | |
| Participants | Patients with a diagnosis of pneumonia | |
| Intervention | ePNA – a CDSS that influences antibiotic choice, site of care, and investigations in pneumonia. | |
| Outcome(s) extracted | O1 – 30-day all-cause mortality  O2 – 7-day readmission to hospital  O3 – Patient disposition (ICU, hospital, home)  O4 – Time to antibiotics in minutes  O5 – Guideline adherent antibiotic prescribing  O6 – Empiric extended spectrum antibiotic use  O7 – Vancomycin use | |
| *Risk of Bias (ROBINS-I)* | | |
| Bias Domain | **Authors’ judgement** | **Supporting information** |
| *Bias due to confounding* | O1 – O7 - Moderate | O1 – O7 - The authors make a reasonable attempt to address most confounding including disease severity, cluster, and secular trends. However, including time of scheduled implementation as a fixed effect is unlikely to provide optimal adjustment for secular trends and some residual or unmeasured confounding is still plausible. |
| *Bias due to selection of participants* | O1 – O7 - Moderate | O1 – O7 - The selection of participants was made on the basis of ICD-10 codes of pneumonia and thoracic imaging findings. It is possible that introduction of an automated pneumonia pop-up in the EHR could influence diagnosis rates thus causing a selection bias. |
| *Bias in classification of interventions* | O1 – O7 - Low | Intervention groups defined based on scheduled implementation time. It is assumed that the scheduled implementation time roughly matches the actual implementation time. |
| *Bias due to deviation from interventions* | O1 – O7 - Low | O1 – O7 – There is no clear evidence of deviation from the intended intervention outside what would be expected in normal clinical practice. |
| *Bias due to missing data* | O1 -O7 - Low | O1 – O7 – Missing data is explicitly reported as being less than 1% and is unlikely to significantly bias results. |
| *Bias in measurements of outcomes* | O1 – O7 - Low | O1 – O7 – All outcomes reported are objective and unlikely to be subject to bias due to non-blinding of outcome assessors. |
| *Bias in selection of reported result* | O1 – O7 - Moderate | O1 – O7 – Study registered with clinicaltrials.gov and outcomes used are in-keeping with these. However, there are multiple post-hoc sensitivity analyses performed and some of these conflict with the findings of primary analysis, in particular the ITS analysis. |
| *Overall risk of bias* | O1 – O7 – Moderate | |
| *Other comments* | Multiple post-hoc sensitivity analyses performed by authors. Only primary analysis results extracted. | |

*CDSS: Clinical Decision Support Software; ICU: Intensive Care Unit; ICD-10: International Classification of Diseases 10^th^ Edition; EHR: Electronic Health Record; ITS: Interrupted Time Series.*

| Demonchy, 2014 | | |
| --- | --- | --- |
| Study design | Controlled before-after study | |
| Participants | Patients with community acquired UTI (cystitis, pyelonephritis, acute prostatitis) | |
| Intervention | The CDSS provided recommendations regarding investigations, indications for hospitalisation, antibiotic treatment and follow up. | |
| Outcome(s) extracted | Guideline adherent antibiotic prescription (choice and duration) | |
| *Risk of Bias (ROBINS-I)* | | |
| Bias Domain | **Authors’ judgement** | **Supporting information** |
| *Bias due to confounding* | Serious | This was a controlled before and after study that adjusted for several confounders in statistical analysis. However, there are confounders that were not accounted for fully including infection severity, patient age, and secular trends. |
| *Bias due to selection of participants* | Serious | This study selected patients on the basis of UTI diagnosis. The CDSS had functionality to support diagnosis of UTI which may cause selection bias. |
| *Bias in classification of interventions* | Low | Intervention status clearly defined and unlikely to bias outcome. |
| *Bias due to deviation from interventions* | Low | No obvious deviations from assignment to CDSS beyond that would be expected in normal clinical practice. |
| *Bias due to missing data* | Serious | Participants with missing data on diagnosis and or antibiotic prescription were excluded. Given that the CDSS influences diagnosis of UTI, this may introduce significant bias. The authors highlight in their discussion that patients with missing data may be the least compliant with guidelines. |
| *Bias in measurements of outcomes* | Low | Outcome measures are extracted from the EHR and are likely to be relatively accurate. Outcome assessors were blinded and outcomes were assessed by 2 independent clinicians. |
| *Bias in selection of reported result* | Moderate | The authors report that there was a predefined protocol, but this was not provided. Outcomes were analysed in multiple separate subgroups. |
| *Overall risk of bias* | Serious | |
| *Other comments* | Authors also reported the use of CDSS and factors associated with its use. | |

*UTI: Urinary Tract Infection; CDSS: Clinical Decision Support Software; EHR: Electronic Health Record.*

| Evans, 1994 | | |
| --- | --- | --- |
| Study design | Uncontrolled before-after study | |
| Participants | Patients being prescribed antibiotics in a private hospital & major teaching centre. | |
| Intervention | ‘Antibiotic consultant’ – this software utilises patient information in the medical record and determines the most likely pathogen and suggests five antibiotic regimens that would be effective. | |
| Outcome(s) extracted | O1 – antibiotic appropriateness  O2 – time to appropriate antibiotic after culture collection  O3 – antibiotic cost | |
| *Risk of Bias (ROBINS-I)* | | |
| Bias Domain | **Authors’ judgement** | **Supporting information** |
| *Bias due to confounding* | O1 + O2 + O3 – Serious | O1 + O2 + O3 – This is an uncontrolled study and there was no adjustment made for confounders such as the infectious syndrome being treated, disease severity, and patient characteristics. |
| *Bias due to selection of participants* | O1 + O2 + O3 – Serious | O1 + O2 + O3 – The results reported only include patients that had a culture ordered. If the introduction of the intervention changed culture ordering rates this could be a cause selection bias. |
| *Bias in classification of interventions* | O1 + O2 + O3 – Low | O1 + O2 + O3 – Classification of intervention status is clearly defined based on access to the intervention at a particular time period. |
| *Bias due to deviation from interventions* | O1 + O2 + O3 – Low | O1 + O2 + O3 - There is no evidence of deviation from the intervention beyond that expected in normal clinical practice. |
| *Bias due to missing data* | O1 + O2 + O3 – Moderate | O1 + O2 + O3 – Of those who were allocated to the use of the clinical decision support tool, prescribing and culture ordering data was only available for 18/28. These were all junior prescribers. However, these prescribers were included in the control and intervention groups, so this is unlikely to introduce serious bias. |
| *Bias in measurements of outcomes* | O1 + O2 + O3 - Moderate | O1 + O2 + O3 – It is unclear whether outcome assessors were blinded to intervention status/ time period. However, outcomes are all objective and therefore this is unlikely to introduce significant bias. |
| *Bias in selection of reported result* | O1 + O2 + O3 – Moderate | O1 + O2 + O3 – No pre-specified analysis plan is reported. |
| *Overall risk of bias* | O1 – Serious  O2 – Serious  O3 - Serious | |
| *Other comments* | Multiple outcomes are reported but most do not represent the intention-to-treat effect of implementing CDSS and are therefore not included. For example, some compare the recommendation provided by the CDSS to actual prescribing.  For meta-analysis of antibiotic appropriateness unadjusted raw values were used. The before access and after access period were used together as the control period. | |

*CDSS: Clinical Decision Support Software.*

| Gohil, 2024a | | |
| --- | --- | --- |
| Study design | Cluster Randomised Controlled Trial | |
| Participants | Patients with a claim code for pneumonia that was present on admission. Patients transferred to the ICU within 2 calendar days were excluded. | |
| Intervention | A CDSS that provided a prompt to encourage narrower spectrum prescribing for patients with a low risk (under 10%) of MDRO pneumonia | |
| Outcome(s) extracted | O1 – length of hospital stay  O2 – time to ICU transfer (days)  O3 – time to escalation of narrow spectrum antibiotics (days)  O4 – empiric extended spectrum antibiotic use (empiric days prescribed)  O5 – vancomycin use (empiric days prescribed)  O6 – antipseudomonal use (empiric days prescribed) | |
| *Risk of Bias (ROB2 - Cluster)* | | |
| Bias Domain | **Authors’ judgement** | **Supporting information** |
| *Bias due to randomization process* | O1 – O6 – Low Risk | O1 – O6 – Hospitals were randomised in pairs of hospitals with similar characteristics. There is no evidence that baseline differences are due to problems with the randomisation process. |
| *Bias due to timing of identification or recruitment of patients* | O1 – O6 – Low Risk | O1 – O6 – Randomisation of hospital pairs was based on data before the baseline period in the trial. All patients with pneumonia included in the trial based on discharge diagnosis. |
| *Bias due to deviations from intended interventions* | O1 – O6 – Low Risk | O1 – O6 – This is an unlikely source of bias. It is not explicitly reported but it is unlikely that participants were made aware of these prompts as individual informed consent was waived. Results were analysed according to assignment to intervention. |
| *Bias due to missing outcome data* | O1 – O6 – Low Risk | O1 – O6 – Data extracted from the EHR with limited missing data reported. |
| *Bias due to measurement of the outcome* | O1 – O6 – Low Risk | O1 – O6 – The outcomes reported were objective and likely to be accurately recorded in the EHR with few systematic differences between intervention and control groups. |
| *Bias due to selection of the reported result* | O1 – O6 – Low Risk | O1 – O6 – Reported analysis and results are in-keeping with a pre-specified statistical analysis plan. |
| *Overall risk of bias* | O1 – O6 – Low Risk | |
| *Other comments* | None | |

*ICU: Intensive Care Unit; CDSS: Clinical Decision Support Software; MDRO: Multi-Drug-Resistant Organism; EHR: Electronic Health Record.*

| Gohil, 2024b | | |
| --- | --- | --- |
| Study design | Cluster Randomised Controlled Trial | |
| Participants | Patients with a claim code for UTI that was present on admission. Patients transferred to the ICU within 2 calendar days were excluded. | |
| Intervention | A CDSS that provided a prompt to encourage narrower spectrum prescribing for patients with a low risk (under 10%) of MDRO UTI | |
| Outcome(s) extracted | O1 – length of hospital stay  O2 – time to ICU transfer (days)  O3 – time to escalation of narrow spectrum antibiotics (days)  O4 – empiric extended spectrum antibiotic use (empiric days prescribed)  O5 – vancomycin use (empiric days prescribed)  O6 – antipseudomonal use (empiric days prescribed) | |
| *Risk of Bias (ROB2 - Cluster)* | | |
| Bias Domain | **Authors’ judgement** | **Supporting information** |
| *Bias due to randomization process* | O1 – O6 – Low Risk | O1 – O6 – Hospitals were randomised in pairs of hospitals with similar characteristics. There is no evidence that baseline differences are due to problems with the randomisation process. |
| *Bias due to timing of identification or recruitment of patients* | O1 – O6 – Low Risk | O1 – O6 – Randomisation of hospital pairs was based on data before the baseline period in the trial. All patients with UTI were included in the trial based on discharge diagnosis. |
| *Bias due to deviations from intended interventions* | O1 – O6 – Low Risk | O1 – O6 – This is an unlikely source of bias. It is not explicitly reported, but it is unlikely that participants were made aware of these prompts as individual informed consent was waived. Results were analysed according to assignment to intervention. |
| *Bias due to missing outcome data* | O1 – O6 – Low Risk | O1 – O6 – Data extracted from the EHR with limited missing data reported. |
| *Bias due to measurement of the outcome* | O1 – O6 – Low Risk | O1 – O6 – The outcomes reported were objective and likely to be accurately recorded in the EHR with few systematic differences between intervention and control groups. |
| *Bias due to selection of the reported result* | O1 – O6 – Low Risk | O1 – O6 – Reported analysis and results are in keeping with a pre-specified statistical analysis plan. |
| *Overall risk of bias* | O1 – O6 – Low Risk | |
| *Other comments* |  | |

*ICU: Intensive Care Unit; CDSS: Clinical Decision Support Software; UTI: Urinary Tract Infection; MDRO: Multi-Drug-Resistant Organism; EHR: Electronic Health Record.*

| Leibovici, 2013 | | |
| --- | --- | --- |
| Study design | Cluster randomised controlled trial. | |
| Participants | Patients suspected of having a bacterial infection. | |
| Intervention | TREAT is a CDSS that uses a causal probabilistic network to provide recommendations for empirical antibiotic prescribing | |
| Outcome(s) extracted | 180-day mortality | |
| *Risk of Bias (ROB2 - Cluster)* | | |
| Bias Domain | **Authors’ judgement** | **Supporting information** |
| *Bias due to randomization process* | Some Concerns | Hospital wards were randomly allocated to intervention or control groups by drawing a random code from an opaque box in original trial. This study included data only from Israel, therefore it only included 6 wards. Given the small number of wards randomised it is plausible that there are imbalances in patient characteristics. |
| *Bias due to timing of identification or recruitment of patients* | High Risk | Whilst ward allocation to intervention was random patients within the cluster were identified through daily chart review and those performing this do not appear to have been blinded. This could have introduced selection bias. |
| *Bias due to deviations from intended interventions* | Some Concerns | Patients being treated were not aware of the intervention. However, doctors prescribing antibiotics were made aware of trial at the time of prescribing which could influence subsequent behaviour. Intention to treat analysis was conducted. |
| *Bias due to missing outcome data* | Low Risk | Data extracted from a likely complete source and no missing data reported. |
| *Bias due to measurement of the outcome* | Low Risk | There is limited reporting surrounding the collection of outcome data, but it was likely collected form the EHR and the outcome is objective and unlikely to be subject to significant bias. |
| *Bias due to selection of the reported result* | High Risk | There is no pre-specified analysis plan and analysis is reported only from Israel. It is not clear why data from other countries included in the original trial is not included. |
| *Overall risk of bias* | High Risk | |
| *Other comments* | Authors also reported per-protocol results and subgroup analysis of patients with documented bacterial infection. | |

*CDSS: Clinical Decision Support Software; EHR: Electronic Health Record.*

| Ng, 2022 | | |
| --- | --- | --- |
| Study design | Interrupted time series | |
| Participants | All patients in hospital | |
| Intervention | CDSS that provided empirical antibiotic prescribing recommendations for 52 infectious syndromes. | |
| Outcome(s) extracted | O1 – Mortality  O2 – Length of stay (age adjusted)  O3.- Tazocin and carbapenem use  O4.- Other broad spectrum antibiotic use  O5 – 3GCR Klebsiella pneumoniae and Escherichia Coli rates  O6 – Clostridium difficile rates  O7 – Carbapenem resistant Pseudomonas aeruginosa rates  O8 – Carbapenem resistant Acinetobacter baumannii rates | |
| *Risk of Bias (ROBINS-I)* | | |
| Bias Domain | **Authors’ judgement** | **Supporting information** |
| *Bias due to confounding* | O1 – O8 - Serious | O1 – O8 - This study uses an interrupted time series analysis that accounts for secular trends. However, analysis is not adjusted for patient level data such as the disease severity and comorbidities. |
| *Bias due to selection of participants* | O1 – O8 - Low | O1 – O8 - The authors use hospital level data for outcomes such as mortality rate, length of stay, antibiotic use, and AMR rates. These are unbiased and not selected on the basis of post baseline variables. |
| *Bias in classification of interventions* | O1 – O8 - Low | O1 – O8 - The authors have a clear record of when the respective interventions were introduced so classification of intervention time periods are likely to be accurate. |
| *Bias due to deviation from interventions* | O1 – O8 -Low | O1 – O8 - There is limited reporting but there is no clear evidence of deviation from the intended interventions. |
| *Bias due to missing data* | O1 – O8 -No information | O1 – O8 - The authors do not clearly report the extent of missing data in this study. |
| *Bias in measurements of outcomes* | O1 – O8 - Low | O1 -O8 - It is unclear how outcomes were assessed but it is likely data were extracted through hospital databases and the outcomes are objective and unlikely prone to such bias. |
| *Bias in selection of reported result* | O1 – O8 - Moderate | O1 – O8 - There is no pre-specified analysis plan and antibiotic use, and antimicrobial resistance outcomes are reported into multiple subgroups. |
| *Overall risk of bias* | O1 – O8 - Serious | |
| *Other comments* |  | |

*CDSS: Clinical Decision Support Software; 3GCR: 3^rd^ generation cephalosporin resistant; AMR: Antimicrobial Resistance.*

| Paul, 2006 | | |
| --- | --- | --- |
| Study design | Cluster randomised controlled trial. | |
| Participants | Patients suspected of having a bacterial infection. | |
| Intervention | TREAT is a CDSS that uses a causal probabilistic network to provide recommendations for empirical antibiotic prescribing | |
| Outcome(s) extracted | O1 – length of hospital stay  O2 – 30-day mortality  O3 – Antibiotic appropriateness  O4 – Direct costs | |
| *Risk of Bias (ROB2 - Cluster)* | | |
| Bias Domain | **Authors’ judgement** | **Supporting information** |
| *Bias due to randomization process* | O1 – O4 – Some Concerns | O1 – O4 – Hospital wards were randomly allocated to intervention or control groups by drawing a random code from an opaque box. In Germany pairs were matched based on specialty. Number of wards randomised is relatively small and this could result in differences in patient characteristics between groups despite randomisation. |
| *Bias due to timing of identification or recruitment of patients* | O1 – O4 – High Risk | O1 – O4 – Whilst ward allocation to intervention was random patients within groups were identified through daily chart review and those performing this do not appear to have been blinded. This could have introduced selection bias for all outcomes. In addition, appropriateness of empirical antibiotics was defined based on in vitro sensitivity testing and patients without microbiological diagnosis were excluded |
| *Bias due to deviations from intended interventions* | O1 – O4 -Some Concerns | O1 – O4 – Patients being treated were not aware of the intervention. However, doctors prescribing antibiotics were made aware of trial at the time of prescribing which could influence subsequent behaviour. Intention to treat analysis was conducted. |
| *Bias due to missing outcome data* | O1 – Some Concerns  O2 – O4 – Low Risk | O1 – There is missing data related to the length of hospital stay and it is plausible that this could be related to the outcome. For example, length of stay may be more likely to missing for shorter stays or where care was transferred.  O2 – O4 – No missing data or negligible amount of missing data for these outcomes. |
| *Bias due to measurement of the outcome* | O1 – O4 – Low Risk | O1 – O4 – There is limited reporting surrounding the collection of outcome data, but it was likely collected form the EHR and these are objective outcomes unlikely to be subject to significant bias. |
| *Bias due to selection of the reported result* | O1 – O4 – High Risk | O1 – O4 – There is no pre-specified analysis and there is evidence of multiple analyses and analyses in multiple subgroups. |
| *Overall risk of bias* | O1 – O4 – High Risk | |
| *Other comments* | The number of wards randomised was small and chance differences in the characteristics of patients on the ward could also account for any observed differences.  Other outcomes that were reported but not included in the data extraction table include other outcomes related to antibiotic costs (observed side effects costs, ecological costs, total antibiotic costs), duration of fever, duration of stay in patients surviving 30 days, 30-day mortality per protocol. | |

*CDSS: Clinical Decision Support Software; EHR: Electronic Health Record.*

| Rohrig, 2008 | | |
| --- | --- | --- |
| Study design | Uncontrolled before-after study | |
| Participants | Patients admitted to the Surgical Intensive Care Unit | |
| Intervention | Clinical decision support to influence empirical antibiotic prescribing that is based on local guidelines adapted for ICU. | |
| Outcome(s) extracted | O1 - Length of stay on ICU  O2 - Mortality in ICU  O3 - Antibiotic appropriateness (adequacy) | |
| *Risk of Bias (ROBINS-I)* | | |
| Bias Domain | **Authors’ judgement** | **Supporting information** |
| *Bias due to confounding* | O1 + O2 + O3 – Serious | O1 + O2 + O3 – Uncontrolled before and after study design with no adjustment for confounders in statistical analysis as 'too few patients'. Numerous baseline confounders could differ between intervention and control groups over time and affect outcome including infectious syndrome and infection severity, patient characteristics, other antimicrobial stewardship measures and policies implemented during this period. |
| *Bias due to selection of participants* | O1 + O2 + O3 – Serious | O1 + O2 + O3 – Selection bias may result as patients are only included if seen on ward round on Tuesdays or Fridays. This selection bias would disproportionately select patients with longer stays. If the intervention reduces length of stay this would cause selection bias as these patients would be less likely to be seen. |
| *Bias in classification of interventions* | O1 + O2 + O3 – Moderate | O1 + O2 + O3 – Interventions classified based on periods of time. However, there is limited reporting of the start date of the intervention. Given the nature of the intervention it is likely to be implemented at a discrete point in time that is easy to measure. Therefore, whilst reporting is limited, on balance this is unlikely to seriously bias the findings. |
| *Bias due to deviation from interventions* | O1 + O2 + O3 – Low | O1 + O2 + O3 - Poor utilisation of CDSS (only around 30%) but this likely reflects actual practice and the effect of interest is of assignment to intervention. |
| *Bias due to missing data* | O1 + O2 + O3 – No information | O1 + O2 + O3 – limited reporting of missing data. |
| *Bias in measurements of outcomes* | O1 + O2 – Low  O3 - Serious | O1 + O2 - There is limited reporting of outcome assessment but given the objective nature of the outcome it is unlikely to be prone to such bias.  O3 - The authors report that outcome assessors were blinded but there is no detail on how this was achieved. There is also no discussion of who assessed the outcomes in each group and whether this was the same for both groups. |
| *Bias in selection of reported result* | O1 + O2 + O3 – Serious | O1 + O2 + O3 – Authors state three comparative analyses carried out to verify any possible influence of the intervention. There is no a priori statistical analysis plan. |
| *Overall risk of bias* | O1 – Serious  O2 – Serious  O3 - Serious | |
| *Other comments* | Two outcomes related to antibiotic appropriateness were reported – ‘adequacy’ and ‘success’. It is unclear how these differed. Adequacy was chosen as it is reported that these assessments were made blind. This was not included in meta-analysis due to unclear reporting.  There are also outcomes reported where the intervention was used compared to where it was not used. These are not reported as this systematic review focuses on the intention-to-treat effect. | |

*ICU: Intensive Care Unit; CDSS: Clinical Decision Support Software.*

**Summary of ROBINS-I Risk of Bias Assessment for Non-randomised Studies Reporting Clinical Outcomes**

*
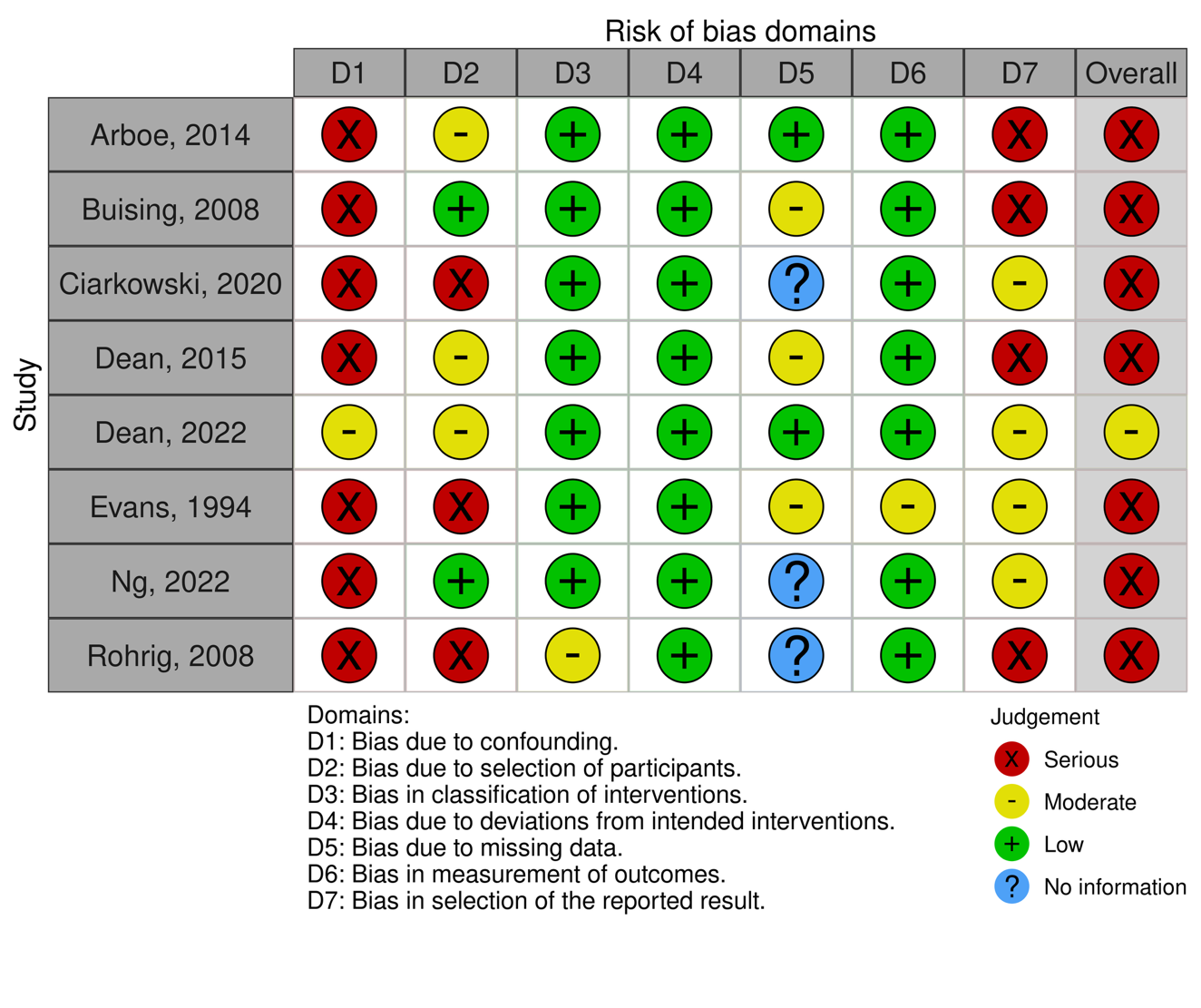
*

The figures presented on page 38-42 represent a summary of the risk of bias assessments performed for individual studies for each outcome domain. As risk of bias assessments were performed for individual outcomes of interest, where discrepant results for two or more outcomes within an outcome domain exist, the outcome with the highest risk of bias was reported in the summary.


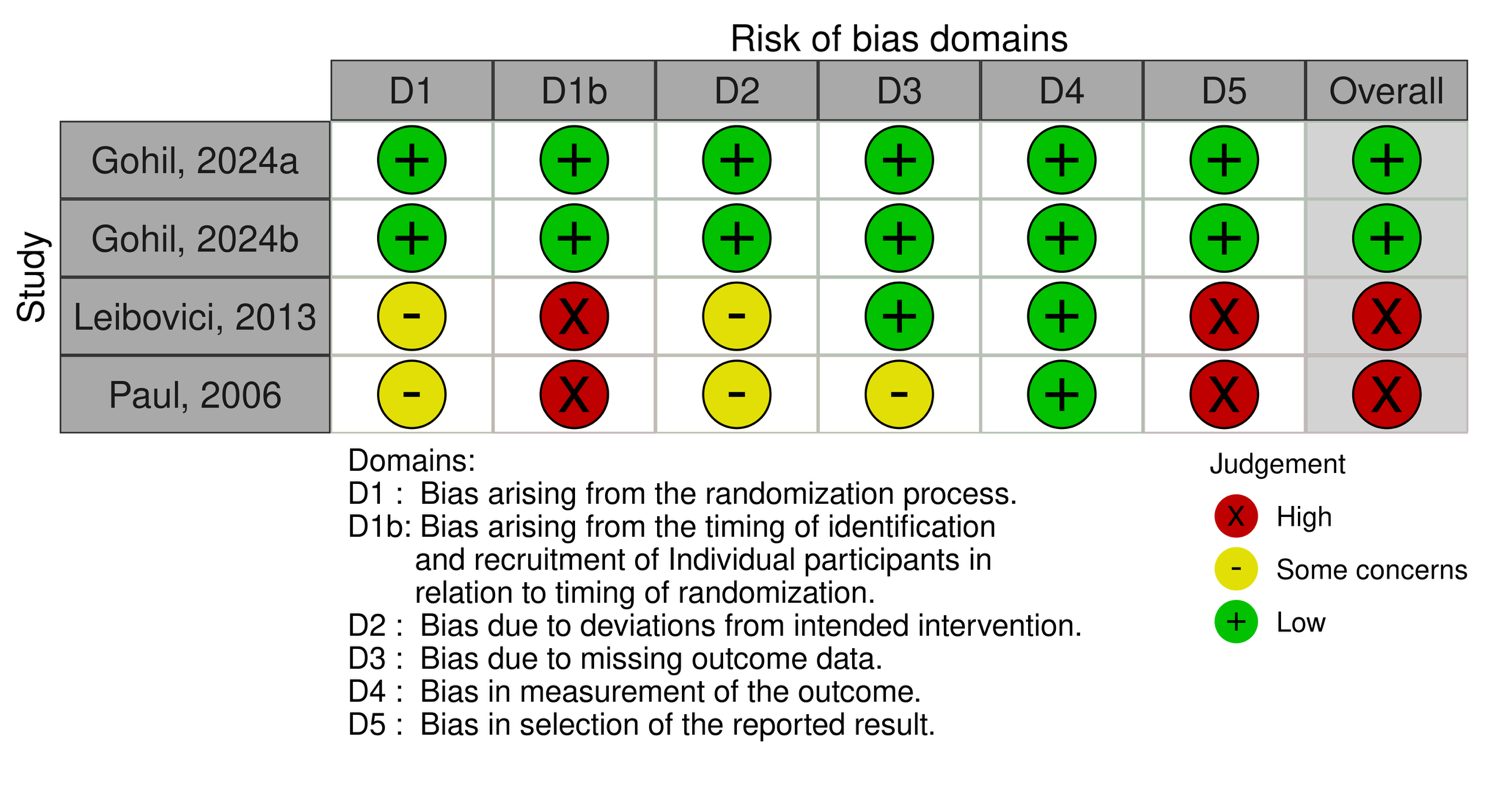
**Summary of Cochrane Risk of Bias 2 Assessment for Randomised Studies Reporting Clinical Outcomes**


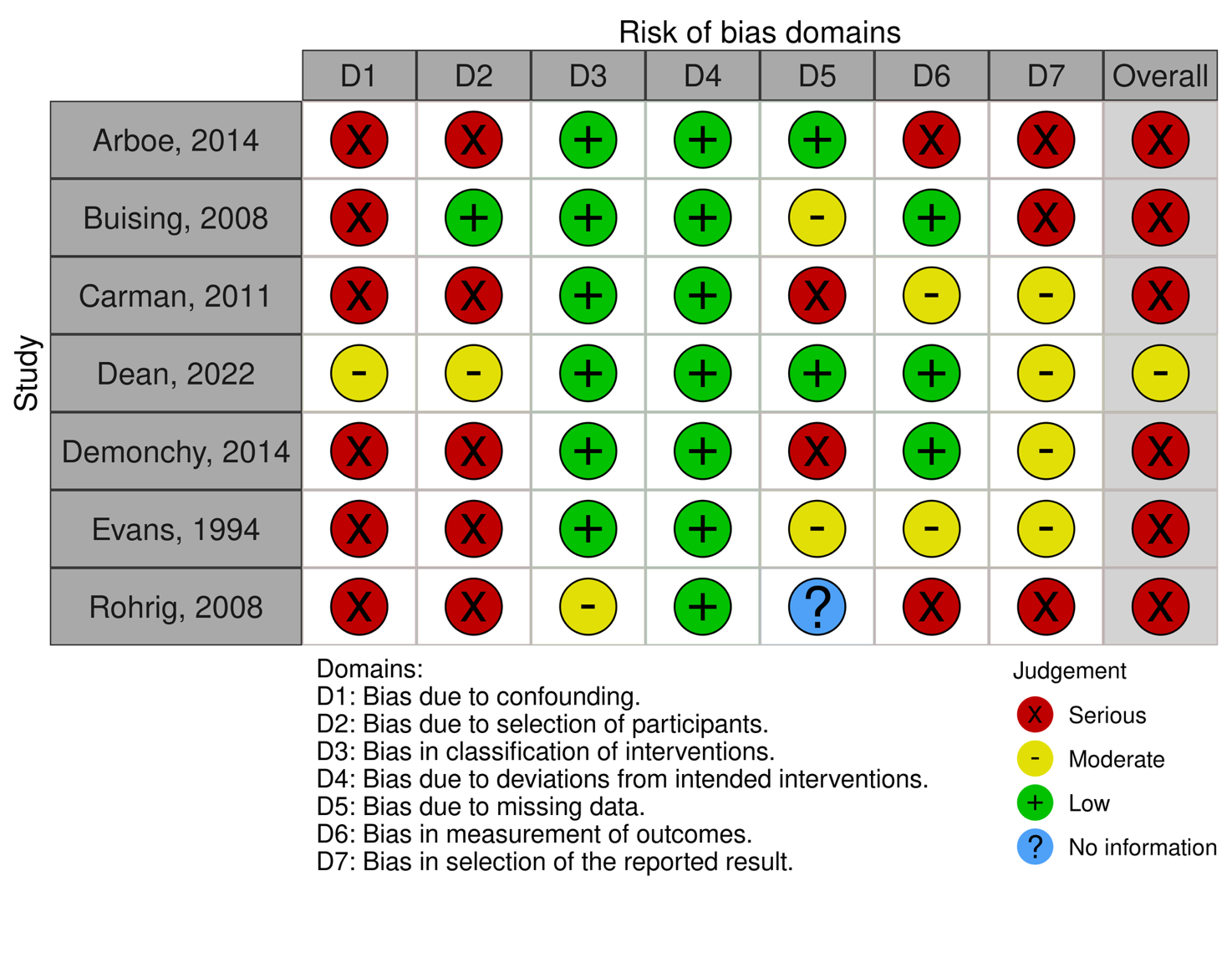
**Summary of ROBINS-I Risk of Bias Assessment for Non-randomised Studies Reporting Antibiotic Appropriateness Outcomes**


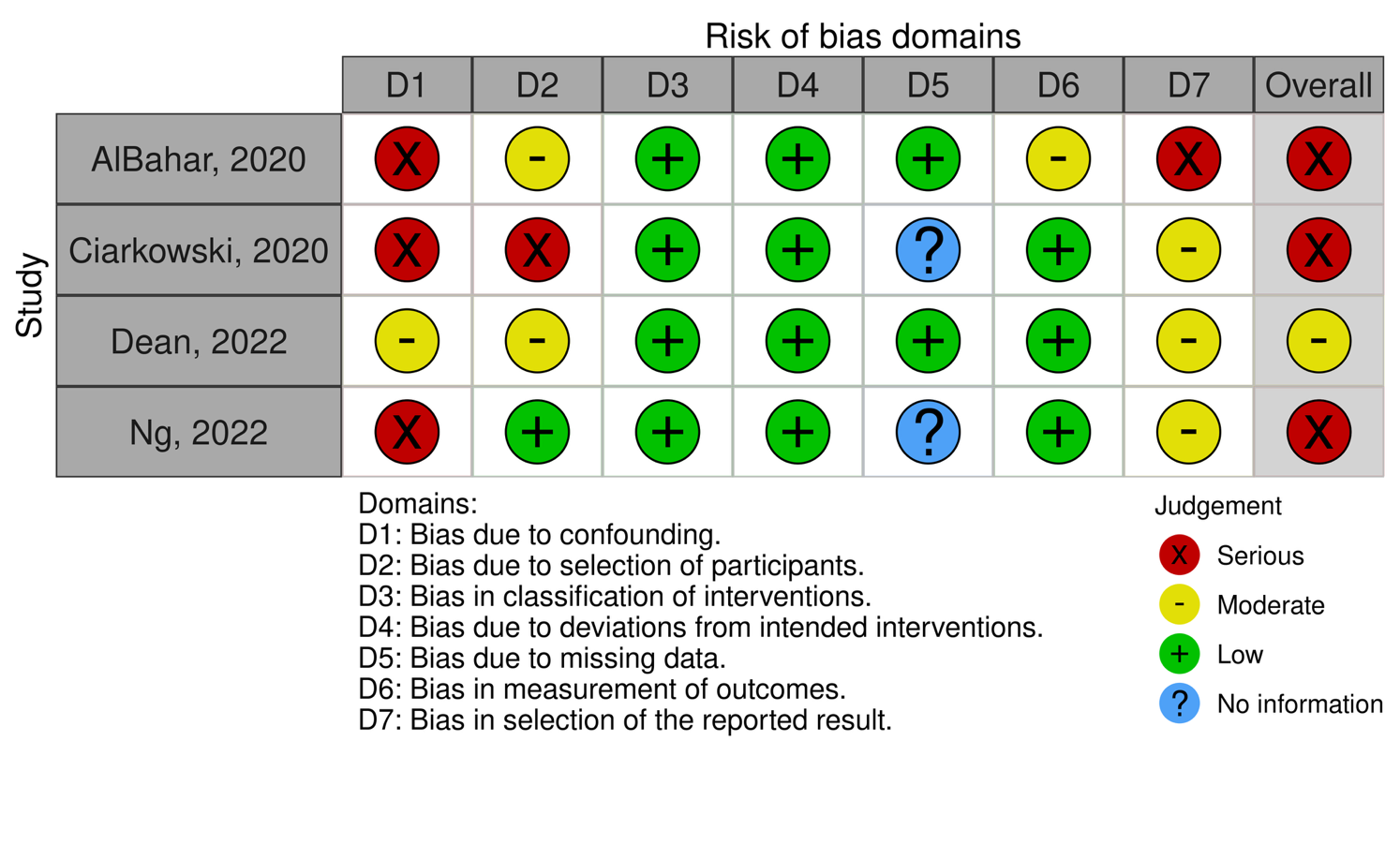
**Summary of ROBINS-I Risk of Bias Assessment for Non-randomised Studies Reporting Antimicrobial Stewardship Outcomes**

**Summary of Cochrane Risk of Bias 2 Assessment for Randomised Studies Reporting Antimicrobial Stewardship Outcomes**


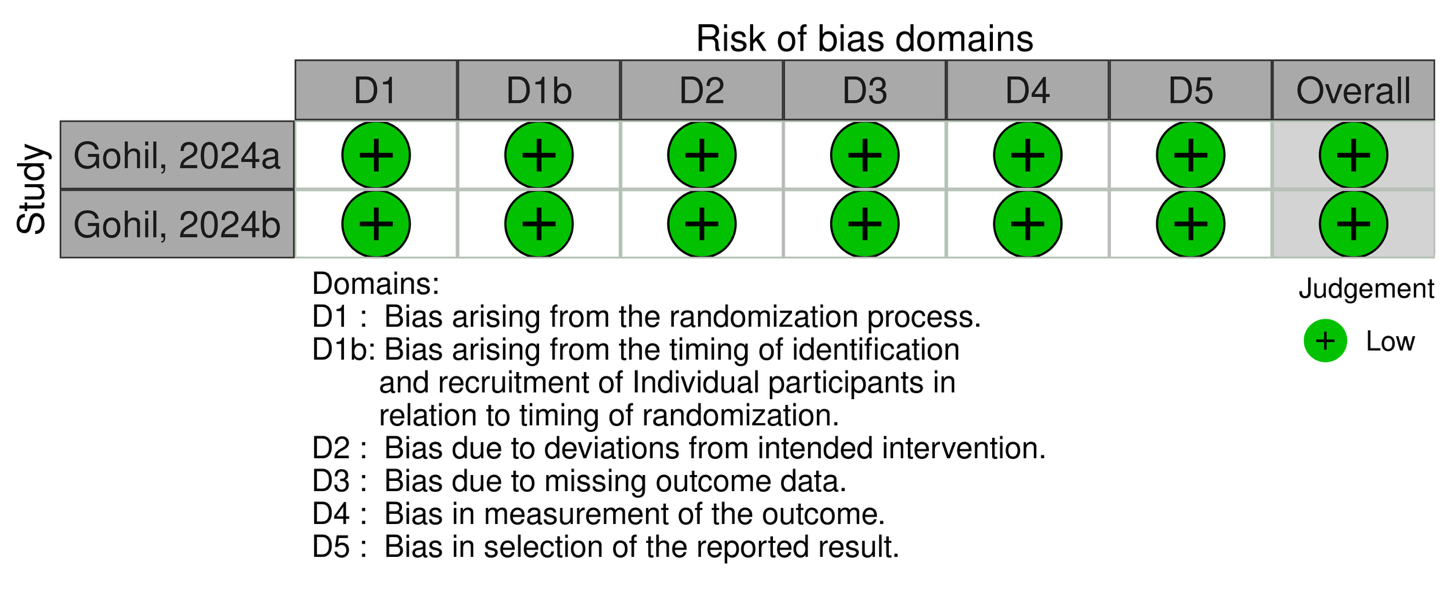


**Supplementary 4 – Mortality Meta-analysis Funnel Plot**


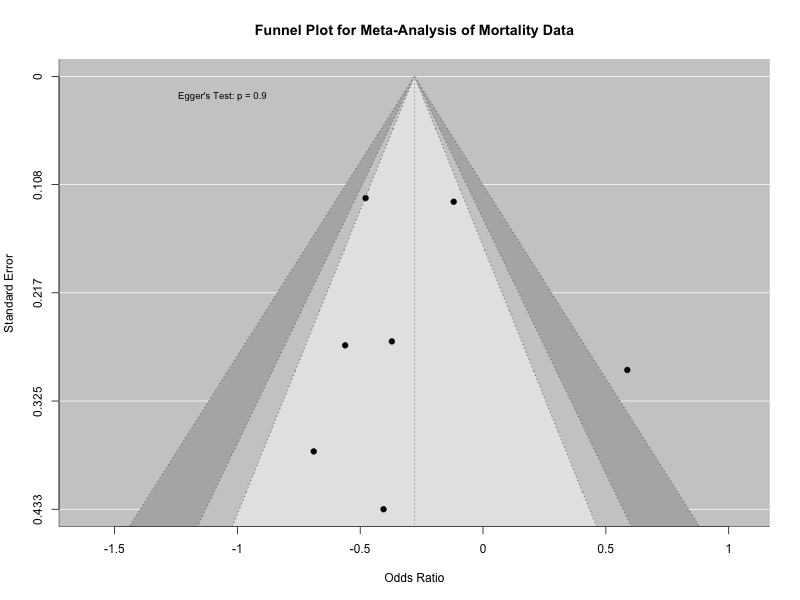


*This figure plots the odds ratio versus the standard error as a funnel plot at the 90% (dark grey), 95% (mid grey), and 99% (light grey) confidence levels. There is no clear evidence of publication bias in this plot. However, the number of studies included is less than that is recommended to make a robust judgement about publication bias using a funnel plot.*

**Supplementary 5 – Summary of Clinical Outcomes**

| Reference | Outcome | Statistical Analysis | Result | Risk of Bias |
| --- | --- | --- | --- | --- |
| Arboe, 2014 | O1 - 30-day mortality (n/N, %)  O2 – length of hospital stay in days (median (range)) | O1 - X^2^ Test  O2 – Not specified | *Outcome 1*  Pre-period (baseline) :40/248 (16.1%)  Post-period (intervention): 26/263 (9.9%)  P = 0.047  *Outcome 2*  Pre-period (baseline): positive microbiology 6.5 (1 – 61), negative microbiology 4 (1 – 60)  Post-period (intervention): positive microbiology 5 (1 – 104), negative microbiology 4 (1 – 46) | O1- Serious  O2 - Serious |
| Buising, 2008 | O1 – time to antibiotics – ED presentation to administration (median (range))  O2 – Mortality* (n (%))  O3 – length of stay in days (median (range))  *Unclear over what time period this outcome is measured. | O1 – ANOVA  O2 - X^2^ Test (adjusted for age)  O3 - ANOVA | *Outcome 1*  Baseline – 171 minutes (15 – 1969 minutes)  Academic detailing – 158 minutes (15 – 1154 minutes)  CDSS – 142 minutes (10 – 1190 minutes)  P < 0.01  *Outcome 2*  Baseline – 37 (9.4%)  Academic detailing – 14 (6.5%)  CDSS – 21 (15.7%)  P = 0.15  *Outcome 3*  Baseline – 4 (1 – 76)  Academic detailing – 4 (1 – 51)  CDSS – 4 (1 – 41)  P = 0.93 | O1 – Serious  O2 – Serious  O3 - Serious |
| Ciarkowski, 2020 | O1 – inpatient mortality (% (95% CI))  O2 – 30-day readmission (% (95% CI))  O3 – length of hospital stay in days (estimated marginal mean (95% CI)) | O1 – 3 – generalised linear models (gamma regression with log link for continuous outcomes, logistic regression for binary outcomes) to compare outcomes of each phase with baseline.  Covariates: age, comorbidities, CURB-65, season of admission | *Outcome 1*  Baseline: 6.28% (95% CI 4.26 - 9.17%)  Education: 6.51% (95% CI 3.31 - 12.41%), P = 0.93  CDSS + stewardship: 4.31% (95% CI 2.2 - 8.27%), P = 0.31  CDSS: 4.28% (95% CI 2.07 - 8.65%), P = 0.35  *Outcome 2*  Baseline: 12.81% (95% CI 9.85 - 16.5)  Education: 10.03% (95% CI 5.98 - 16.34), P = 0.39  CDSS + stewardship: 13.73% (95% CI 9.03 - 20.33), P = 0.78  CDSS: 12.39% (95% CI 7.91 - 18.9), P = 0.90  *Outcome 3*  Baseline: 4.94 (4.53 - 5.39)  Education: 4.54 (3.87 - 5.33), P = 0.37  CDSS + stewardship: 4.46 (3.87 - 5.13), P =0.21  CDSS: 4.68 (4.03 - 5.43), P = 0.54 | O1 – Serious  O2 – Serious  O3 - Serious |
| Dean, 2015 | O1 – 30-day mortality (OR (95% CI))  OR <1 suggests lower mortality in intervention group.  O2 – Length of hospital stay in days (median (95% CI))  O3 – Readmission to hospital (% change in baseline to post-deployment)  O4 – guideline concordant patient disposition (% (95% CI)) | O1 – mixed effects model to account for variation in pneumonia severity over years, differences in event rates, eCURB, HCAP, PaO2/FiO2, pleural effusion, sex. Multiple adjustment strategies tested.  O2- not clearly specified  O3 – not clearly specified  O4 – not clearly specified. | *Outcome 1*  OR 0.69 (95% CI 0.41 – 1.16)  *Outcome 2*  Intervention baseline: 3.0 (95% CI 2.8 – 3.2)  Intervention post-deployment: 2.9 (95% CI 2.8 – 3.0)  Control baseline: 3.1 (95% CI 2.9 – 3.2)  Control second period: 3.0 (95% CI 2.8 – 3.1)  *Outcome 3*  Intervention: - 1.9%  Control: 1.7%  *Outcome 4 - guideline concordant admission*  Intervention baseline: 79% (76 - 82%)  Intervention post-deployment: 84% (82 - 87%)  Control baseline: 82% (79 - 85%)  Control second period: 81% (78 - 84%)  *Outcome 4 – guideline concordant disposition*  *Concordant Discharge*  Intervention baseline: 69% (65 - 73%)  Intervention post-deployment: 71% (67 - 75%)  Control baseline: 72% (68 - 77%)  Control post-deployment: 71% (66 - 75%)  *Concordant Admission*  Intervention baseline: 79% (76 – 82%)  Intervention post-deployment: 84% (82 – 87%)  Control baseline: 82% (79 – 85%)  Control post-deployment: 81% (78 – 84%) | O1 – Serious  O2 – Serious  O3 – Serious  O4 - Serious |
| Dean, 2022 | O1 – 30-day all-cause mortality (OR (95% CI))  ORs <1 suggests lower mortality in intervention group.  O2 – 7-day readmission to hospital (OR (95% CI))  ORs >1 suggests higher rate of readmissions in intervention group.  O3 – patient disposition (% guideline adherent)  O4 – time to antibiotics in minutes (mean (95% CI)) | O1 – Mixed effects model with scheduled implementation time as fixed effect and cluster as a random and fixed effect with validated severity adjustors.  O2 – Not clearly specified. Result is adjusted for severity.  O3 – Mixed effects model as above.  O4 – Not clearly specified. | *Outcome 1*  OR 0.62 (95% CI 0.49 – 0.79), P < 0.001  *Outcome 2*  OR 1.2 (95% CI 0.84 – 1.71), P = 0.31  *Outcome 3*  Outpatient disposition: 29.2% control vs. 46.9% intervention  ICU disposition: 13.5% control vs. 6.1% intervention  Hospital ward disposition: 57.3% control vs. 47% intervention.  P = 0.036  *Outcome 4*  Control: 159.4 (95% CI 156.9 – 161.9)  Intervention: 150.9 (95% CI 144.1 – 157.8)  P < 0.001 | O1 – Moderate  O2 – Moderate  O3 – Moderate  O4 - Moderate |
| Gohil 2024a | O1 – length of hospital stay in days (Difference in differences hazard ratio (95% CI))  O2 – time to ICU transfer in days (Difference in differences hazard ratio (95% CI))  O3 – time to escalation of narrow spectrum antibiotics in days (Difference in differences hazard ratio (95% CI)) | O1 – 3 – proportional hazards model with random effects to account for clustering by hospital and period. | *Outcome 1*  HR 0.96 (95% CI 0.91 – 1.01)  P = 0.13  *Outcome 2*  HR 1.04 (95% CI 0.89 – 1.21)  P = 0.62  *Outcome 3*  HR 0.82 (95% CI 0.69 – 0.97)  P = 0.02 | O1 – Low  O2 – Low  O3 – Low |
| Gohil 2024b | O1 – length of hospital stay in days (Difference in differences hazard ratio (95% CI))  O2 – time to ICU transfer in days (Difference in differences hazard ratio (95% CI))  O3 – time to escalation of narrow spectrum antibiotics in days (Difference in differences hazard ratio (95% CI)) | O1 – 3 – proportional hazards model with random effects to account for clustering by hospital and period. | *Outcome 1*  HR 0.96 (95% CI 0.91 – 1.01)  P = 0.21  *Outcome 2*  HR 0.98 (95% CI 0.85 – 1.12)  P = 0.77  *Outcome 3*  HR 1.03 (95% CI 0.89 – 1.19)  P = 0.66 | O1 – Low  O2 – Low  O3 – Low |
| Leibovici, 2013 | Mortality (180-day) | Wilcoxin test | Intervention: 27%  Control: 29%  P = 0.2 | High |
| Ng, 2022 | O1 – inpatient mortality rate per 100 discharges per month (estimate (95% CI))  O2 – age adjusted length of stay in days per month (estimate (95% CI)) | Segmented regression for level and trend changes after each intervention. Only trend changes reported for these outcomes. | *Outcome 1*  Intervention 1 (prescription review and feedback): “stable”  Intervention 2 (compulsory use of CDSS): “stable”  Intervention 3 (mixed compulsory and voluntary CDSS use): -0.05 (95% CI-0.08 to – 0.03)  *Outcome 2*  Intervention 1 (prescription review and feedback): 0.03 (0.004 – 0.05)  Intervention 2 (compulsory use of CDSS): 0.01 (0.005 – 0.016)  Intervention 3 (mixed compulsory and voluntary CDSS use): -0.04 (-0.06 - -0.03) | O1 – Serious  O2 - Serious |
| Paul, 2006 | O1 – length of hospital stay in days (mean (SD))  O2 – 30-day mortality (%, N) | O1 – Mann Whitney U Test  O2 – Pearson X^2^ and combined analysis with Mantel-Haenszel statistic adjusting for recruitment site. | *Outcome 1*  Intervention: Mean 8.83 (SD 11.29)  Control: 9.45 (SD 11.52)  P = 0.055  *Outcome 2*  Intervention: 12.9%, N = 1153  Control: 14.3%, N = 1012  P = 0.611 | O1 – High  O2 - High |
| Rohrig, 2008 | O1 – Length of ICU stay in hours (mean (SD))  O2 – Mortality in ICU (n (%)) | O1 – Mann-Whitney U Test  O2 – X^2^ Test | *Outcome 1*  Pre-period: 472 hours (SD 538)  Post-period: 337 hours (SD 275)  P = 0.07  *Outcome 2*  Pre-period: 18 (41.9%)  Post-period: 30 (26.5%)  P = 0.06 | O1 – Serious  O2 – Serious |

*Supplementary 5 presents the clinical outcomes extracted from individual studies in this systematic review. CDSS: Clinical Decision Support Software; ED: Emergency Department; ANOVA: Analysis of Variance; OR: Odds Ratio; HCAP: Health Care Associated Pneumonia; PaO2: Partial Pressure of Oxygen; FiO2: Fraction of Inspired Oxygen; ICU: Intensive Care unit; HR: Hazard Ratio; MDRO: Multi-Drug-Resistant Organism; SD: Standard Deviation; CI: Confidence Interval. CURB-65 and eCURB refer to severity scoring systems used in pneumonia.*

**Supplementary 6 – Summary of Antibiotic Appropriateness Outcomes**

| Reference | Outcome | Statistical Analysis | Result | Risk of Bias |
| --- | --- | --- | --- | --- |
| Arboe, 2014 | O3 – appropriate coverage of organisms (n (%)) | Unclear if this comparison made statistically. | *Outcome 3*  Pre-period (baseline): 41/ 80 (51.3%)  Post-period (intervention): 51/ 82 (62.2%) | O2 - Serious |
| Buising, 2008 | O4 – appropriate coverage of likely organisms (atypical and typical) (Odds ratio (95% CI))  OR >1 suggests appropriate coverage more likely during intervention period. | Multivariable logistic regression model  Covariates: disease severity, age, suspected aspiration | *Outcome 4*  OR 1.99 (95% CI 1.07 – 3.69)  P = 0.02 | O4- Serious |
| Carman, 2011 | Guideline adherent empiric antibiotics (% guideline adherent, OR)  OR >1 suggests appropriate coverage more likely during intervention period. | Logistic regression  Covariates: age & gender | Pre-period: 86.8%  Post-period (week 6): 90.6%  Post-period (week 12): 96.7%  OR 2.41(intervention versus control)  P = 0.000  No confidence intervals provided. | Serious |
| Dean, 2022 | O5– appropriateness of antibiotic (guideline adherent) (OR (95% CI))  OR >1 suggests appropriate coverage more likely during intervention period. | O5 – not clearly specified. Outcome adjusted for severity. | *Outcome 5*  OR 1.9 (95% CI 1.54 – 2.30), P < 0.001 | O5 - Moderate |
| Demonchy, 2014 | Guideline concordant antibiotics - agent and duration (Odds ratio ( 95% CI))  OR >1 suggests appropriate coverage more likely during intervention period. | Multivariate logistic mixed effect models.  Random effects – hospital  Fixed effects – CDDS use, doctor seniority, treatment time (weekday/ weekend/ nights), diagnosis | OR 1.07 (95% CI 0.69 – 1.66)  P = 0.77 | Serious |
| Evans, 1994 | O1 – antibiotic appropriateness based on in vitro susceptibility (%)  O2 – time to appropriate antibiotic after culture collection (mean hours) | Methods and results from statistical comparisons not clearly reported. | *Outcome 1*  Control (pre-intervention): 64%  Intervention: 67%  Control (post-intervention): 67%  *Outcome 2*  Control (pre-intervention): 22 hours  Intervention: 16 hours  Control (post-intervention): 19 hours  P-value not reported, authors state non-significant. | O1 – Serious  O2 - Serious |
| Paul, 2006 | O3 – appropriateness of antibiotic based on in-vitro susceptibility (OR (95% CI))  OR >1 suggests appropriate coverage more likely during intervention period. | Generalised estimating equation approach with logistic regression model with exchangeable correlations adjusting for medical centre and accounting for clustering by ward. | *Outcome 3*  OR 1.48 (95% CI 0.95 – 2.29)  P = 0.082 | O3 - Serious |
| Rohrig, 2008 | O3 – “Adequate Therapy” (mean % adequate (95% CI)) | O3 – Mann-Whitney U Test | *Outcome 3*  Pre-period: 47.8% (95% CI: 38.3% – 57.2%)  Post-period: 66.5% (95% CI: 60.8% - 72.2%)  P = < 0.01 | O3 - Serious |

*Supplementary 6 presents the antibiotic appropriateness outcomes extracted from individual studies in this systematic review. CI: Confidence Interval; OR: Odds Ratio.*

**Supplementary 7 – Summary of Antimicrobial Stewardship Outcomes**

| Reference | Outcome | Statistical Analysis | Result | Risk of Bias |
| --- | --- | --- | --- | --- |
| Al Bahar, 2020 | O1 – Total antibiotic usage  O2 – Penicillin usage  O3 – Carbapenem usage  O4 – Cephalosporin usage  All outcomes expressed as mean difference in Defined Daily Dose (DDD) per 1000 bed-days. Positive values suggest higher rates of prescribing during the period without structured prescribing. | Student’s t-test to compare DDD/1000 bed-days during and after intervention. | *Outcome 1*  110 DDD/1000 bed-days  P = 0.026  *Outcome 2*  28 DDD/1000 bed-days  P = 0.229  *Outcome 3*  15 DDD/1000 bed-days  P < 0.001  *Outcome 4*  8 DDD/1000 bed-days  P < 0.001  Note – no confidence intervals reported | O1 – Serious  O2 – Serious  O3 – Serious  O4 - Serious |
| Ciarkowski, 2020 | O4 – length of IV antibiotics (estimated marginal mean (95% CI))  O5 – total length of antibiotics (estimated marginal mean (95% CI)) | O1 – 3 – generalised linear models (gamma regression with log link for continuous outcomes, logistic regression for binary outcomes) to compare outcomes of each phase with baseline.  Covariates: age, comorbidities, CURB-65, season of admission | *Outcome 4*  Baseline: 3.49 (3.19 - 3.82)  Education: 3.16 (2.68 - 3.74), P =0.32  CDSS + stewardship: 2.73 (2.36 - 3.16), P = <0.01  CDSS: 2.81 (2.41 - 3.28), P =0.02  *Outcome 5*  Baseline: 6.4 (5.97 - 6.86)  Education: 6.04 (5.33 - 6.86), P = 0.45  CDSS + stewardship: 5.54 (4.96 - 6.19), P = 0.03  CDSS: 5.98 (5.31 - 6.73), P =0.34 | O4 – Serious  O5 - Serious |
| Dean, 2022 | O6 – empiric extended spectrum use  O7 – vancomycin use (all antibiotics active against MRSA) | O6 – 7 – not clearly specified. | *Outcome 6*  OR 0.88 (95% CI 0.75 – 1.04), P = 0.14  *Outcome 7*  Control 13% vs. intervention 10%, P < 0.001 | O6 – Moderate  O7 - Moderate |
| Gohil, 2024a | O4 – empiric extended spectrum antibiotic use - empiric days prescribed (Difference in Differences rate ratio (95% CI))  O5 – vancomycin use - empiric days prescribed (Difference in Differences rate ratio (95% CI))  O6 – antipseudomonal use - empiric days prescribed (Difference in Differences rate ratio (95% CI))  RRs <1 suggests that there was a greater reduction in antibiotic use between baseline and intervention periods in the CDSS group compared to the routine stewardship group. | O4 -6 – generalised linear mixed effects models with clustering in hospital and time period as random effects. | *Outcome 4*  RR 0.72 (95% CI 0.66 – 0.78)  P < 0.001  *Outcome 5*  RR 0.77 (95% CI 0.71 – 0.83)  P < 0.001  *Outcome 6*  RR 0.68 (95% CI 0.61 – 0.75)  P <0.001 | O4 – Low  O5 – Low  O6 - Low |
| Gohil, 2024b | O4 – empiric extended spectrum antibiotic use - empiric days prescribed (Difference in Differences rate ratio (95% CI))  O5 – vancomycin use - empiric days prescribed (Difference in Differences rate ratio (95% CI))  O6 – antipseudomonal use - empiric days prescribed (Difference in Differences rate ratio (95% CI))  RRs <1 suggests that there was a greater reduction in antibiotic use between baseline and intervention periods in the CDSS group compared to the routine stewardship group. | O4 -6 – generalised linear mixed effects models with clustering in hospital and time period as random effects. | *Outcome 4*  RR 0.83 (95% CI 0.77 – 0.89)  P < 0.001  *Outcome 5*  RR 0.89 (95% CI 0.82 – 0.96)  P = 0.002  *Outcome 6*  RR 0.79 (95% CI 0.72 – 0.87)  P < 0.001 | O4 – Low  O5 – Low  O6 - Low |
| Ng, 2022 | O3 – Tazocin and carbapenem use - DDD per 1000 patient days per month (Estimate (95% CI))  O4 – Other broad-spectrum use - DDD per 1000 patient days per month (Estimate (95% CI))  O5 – 3GCR Klebsiella Pneumoniae and Escherichia Coli - number of clinical isolates per 1000 inpatient days (Estimate (95% CI))  O6 – Clostridium Difficile -number of clinical isolates per 1000 inpatient days (Estimate (95% CI))  O7 – Carbapenem resistant Pseudomonas Aeruginosa - number of clinical isolates per 1000 inpatient days (Estimate (95% CI))  O8 – Carbapenem resistant Acinetobacter baumannii - number of clinical isolates per 1000 inpatient days (Estimate (95% CI)) | Segmented regression for level and trend change after each intervention. | *Outcome 3*  Intervention 1 (prescription review and feedback):  Level change: -6.01(-9.82 - -2.20), P < 0.002  Trend change: -0.84 (-1.06 - -0.62), P < 0.001  Intervention 2 (compulsory use of CDSS):  Level change: 8.45 (2.82 to 14.08), p = 0.004  Trend change: −0.55 (−0.74 to −0.36), p = < 0.001  Intervention 3 (mixed compulsory and voluntary CDSS use):  Level change: 8.29 (2.63 to 13.94) p = 0.004  Trend change: 0.50 (0.21 to 0.79), p = 0.001  *Outcome 4*  Intervention 1 (prescription review and feedback):  Level change: 103.46 (49.23 to 157.68), p < 0.001  Trend change: −11.05(−15.55 to −6.55, p < 0.001  Intervention 2 (compulsory use of CDSS):  Level change: −50.78 (−121.8 to 20.24), p = 0.16  Trend change: 9.00 (4.75 to 13.25), p < 0.001  Intervention 3 (mixed compulsory and voluntary CDSS use):  Level change: 109.2 (57.79 to 160.61), p = < 0.001  Trend change: 1.31 (−0.99 to 3.61), p = 0.26  *Outcome 5*  Intervention 1 (prescription review and feedback):  Level change: - 0.55 (-1.11 to 0.02), p = 0.06  Trend change: 0.03 (−0.01 to 0.06), p = 0.19  Intervention 2 (compulsory use of CDSS):  Level change: 0.70 (0.29 to 1.10), p = 0.001  Trend change: -0.001 (−0.03 to 0.03), p = 0.93  Intervention 3 (mixed compulsory and voluntary CDSS use):  Level change: 0.02 (-0.42 to 0.45), p = 0.94  Trend change: 0.02 (−0.03 to 0.06), p = 0.40  *Outcome 6*  Intervention 1 (prescription review and feedback):  Level change: 0.08 (−0.08 to 0.24), p = 0.33  Trend change: 0.037 (0.02 to 0.05), p < 0.001  Intervention 2 (compulsory use of CDSS):  Level change: 0.08 (−0.04 to 0.19), p = 0.19  Trend change: −0.02 (−0.03 to −0.01), p < 0.001  Intervention 3 (mixed compulsory and voluntary CDSS use):  Level change: −0.03 (−0.12 to 0.05), p = 0.42  Trend change: 0.0017 (−0.003 to 0.010), p = 0.49  *Outcome 7*  Intervention 1 (prescription review and feedback):  Level change: −0.11 (−0.22 to −0.01), p = 0.03  Trend change: 0.002 (−0.01 to 0.01), p = 0.57  Intervention 2 (compulsory use of CDSS):  Level change: 0.01 (−0.06 to 0.09), p = 0.66  Trend change: −0.0005 (−0.004 to 0.003), p = 0.76  Intervention 3 (mixed compulsory and voluntary CDSS use):  Level change: -0.04 (−0.09 to 0.01), p = 0.14  Trend change: 0.006 (0.002 to 0.01), p = 0.007  *Outcome 8*  Intervention 1 (prescription review and feedback):  Level change: 0.17 (−0.10 to 0.45), p = 0.21  Trend change: −0.01 (−0.03 to 0.01), p = 0.20  Intervention 2 (compulsory use of CDSS):  Level change: 0.04 (−0.21 to 0.28), p = 0.77  Trend change: 0.004 (−0.01 to 0.02), p = 0.69  Intervention 3 (mixed compulsory and voluntary CDSS use):  Level change: 0.04 (−0.06 to 0.13), p = 0.45  Trend change: -0.002 (−0.01 to 0.002), p = 0.33 | O3 – Serious  O4 – Serious  O5 – Serious  O6– Serious  O7 – Serious  O8 – Serious |

*Supplementary 7 presents the antimicrobial stewardship outcomes extracted from individual studies in this systematic review. DDD: Defined Daily Dose; IV: Intravenous; CI: Confidence Interval; CDSS: Clinical Decision Support Software; OR: Odds Ratio; MRSA: Methicillin-Resistant Staphylococcus Aureus; RR: Rate Ratio; 3GCR: 3^rd^ Generation Cephalosporin Resistance. CURB-65 is a scoring system used to stratify pneumonia severity.*

**Supplementary 8 – Summary of Health Economic Outcomes**

| Reference | Outcome | Statistical Analysis | Result | Risk of Bias |
| --- | --- | --- | --- | --- |
| Arboe, 2014 | O4 – Antibiotic cost (Direct costs in € per day, median (range)) | Not reported | *Outcome 4*  Pre-period (baseline): 1.4 (0 – 35)  Post-period (intervention): 1.4 (0 – 24) | O4 - Serious |
| Buising, 2008 | O5 – average cost of antibiotics per patient | Not reported | *Outcome 5*  Baseline - $72.07  Academic detailing - $94.47  CDSS - $ 84.04 | O5 - Serious |
| Ciarkowski, 2020 | O6 – Total cost per visit converted to 2015 US dollars and normalised to baseline cost (cost (95% CI)) | Gamma regression adjusted for age, Charlson Comorbidity Index, CURB-65, and flu-season. | *Outcome 6*  Baseline – 1 (0.9 – 1.12)  Education: 0.79 (0.65 – 0.97), p = 0.05  CDSS + stewardship: 0.80 (0.67 – 0.95), p = 0.03  CDSS: 0.84 (0.69 – 1.01), p = 0.11 | O6 - Serious |
| Evans, 1994 | O3 – Antibiotic Cost (mean cost for 24 hours therapy in $) | Students t-test | *Outcome 3*  Control (pre-intervention): 49.39  Intervention: 48.06  Control (post-intervention): 44.95  No significant difference. | O3 - Serious |
| Paul, 2006 | O4 – Direct costs in Euros per patient, mean (SD) | Mann-Whitney U test. | *Outcome 4*  Control: 37.9 (54.2)  Intervention: 40.2 (57.6)  P = 0.473 | O4 - High |

*Supplementary 8 presents the health economic outcomes extracted from individual studies in this systematic review. CDSS: Clinical Decision Support Software; US: United States; CI: Confidence Interval; SD: Standard Deviation. CURB-65 is a scoring system used to stratify pneumonia severity.*

**Supplementary 9 – PRISMA Checklist**

| **Section and Topic** | **Item #** | **Checklist item** | **Location where item is reported** |
| --- | --- | --- | --- |
| **TITLE** | | |  |
| Title | 1 | Identify the report as a systematic review. | 1 |
| **ABSTRACT** | | |  |
| Abstract | 2 | See the PRISMA 2020 for Abstracts checklist. | 2-3 |
| **INTRODUCTION** | | |  |
| Rationale | 3 | Describe the rationale for the review in the context of existing knowledge. | 4-5 |
| Objectives | 4 | Provide an explicit statement of the objective(s) or question(s) the review addresses. | 5 |
| **METHODS** | | |  |
| Eligibility criteria | 5 | Specify the inclusion and exclusion criteria for the review and how studies were grouped for the syntheses. | 6-7, Table 1 |
| Information sources | 6 | Specify all databases, registers, websites, organisations, reference lists and other sources searched or consulted to identify studies. Specify the date when each source was last searched or consulted. | 7, Supplementary 1 |
| Search strategy | 7 | Present the full search strategies for all databases, registers and websites, including any filters and limits used. | Supplementary 1 |
| Selection process | 8 | Specify the methods used to decide whether a study met the inclusion criteria of the review, including how many reviewers screened each record and each report retrieved, whether they worked independently, and if applicable, details of automation tools used in the process. | 7 |
| Data collection process | 9 | Specify the methods used to collect data from reports, including how many reviewers collected data from each report, whether they worked independently, any processes for obtaining or confirming data from study investigators, and if applicable, details of automation tools used in the process. | 8-9 |
| Data items | 10a | List and define all outcomes for which data were sought. Specify whether all results that were compatible with each outcome domain in each study were sought (e.g. for all measures, time points, analyses), and if not, the methods used to decide which results to collect. | 8-9 |
|  | 10b | List and define all other variables for which data were sought (e.g. participant and intervention characteristics, funding sources). Describe any assumptions made about any missing or unclear information. | 8-9 |
| Study risk of bias assessment | 11 | Specify the methods used to assess risk of bias in the included studies, including details of the tool(s) used, how many reviewers assessed each study and whether they worked independently, and if applicable, details of automation tools used in the process. | 9 |
| Effect measures | 12 | Specify for each outcome the effect measure(s) (e.g. risk ratio, mean difference) used in the synthesis or presentation of results. | 9-10 |
| Synthesis methods | 13a | Describe the processes used to decide which studies were eligible for each synthesis (e.g. tabulating the study intervention characteristics and comparing against the planned groups for each synthesis (item #5)). | 9-10 |
|  | 13b | Describe any methods required to prepare the data for presentation or synthesis, such as handling of missing summary statistics, or data conversions. | 9-10 |
|  | 13c | Describe any methods used to tabulate or visually display results of individual studies and syntheses. | 9-10 |
|  | 13d | Describe any methods used to synthesize results and provide a rationale for the choice(s). If meta-analysis was performed, describe the model(s), method(s) to identify the presence and extent of statistical heterogeneity, and software package(s) used. | 9 |
|  | 13e | Describe any methods used to explore possible causes of heterogeneity among study results (e.g. subgroup analysis, meta-regression). | 10 |
|  | 13f | Describe any sensitivity analyses conducted to assess robustness of the synthesized results. | None reported due to small number of studies. |
| Reporting bias assessment | 14 | Describe any methods used to assess risk of bias due to missing results in a synthesis (arising from reporting biases). | 10 |
| Certainty assessment | 15 | Describe any methods used to assess certainty (or confidence) in the body of evidence for an outcome. | 10 |
| **RESULTS** | | |  |
| Study selection | 16a | Describe the results of the search and selection process, from the number of records identified in the search to the number of studies included in the review, ideally using a flow diagram. | 11-12, Figure 1 |
|  | 16b | Cite studies that might appear to meet the inclusion criteria, but which were excluded, and explain why they were excluded. | 11-12 |
| Study characteristics | 17 | Cite each included study and present its characteristics. | 12-13, Table 2 |
| Risk of bias in studies | 18 | Present assessments of risk of bias for each included study. | Supplementary 3 |
| Results of individual studies | 19 | For all outcomes, present, for each study: (a) summary statistics for each group (where appropriate) and (b) an effect estimate and its precision (e.g. confidence/credible interval), ideally using structured tables or plots. | Supplementary 5-8 |
| Results of syntheses | 20a | For each synthesis, briefly summarise the characteristics and risk of bias among contributing studies. | 17-21 |
|  | 20b | Present results of all statistical syntheses conducted. If meta-analysis was done, present for each the summary estimate and its precision (e.g. confidence/credible interval) and measures of statistical heterogeneity. If comparing groups, describe the direction of the effect. | 17-21, Figure 3-5 |
|  | 20c | Present results of all investigations of possible causes of heterogeneity among study results. | 17-21, Figure 3-5 |
|  | 20d | Present results of all sensitivity analyses conducted to assess the robustness of the synthesized results. | Sensitivity analyses not conducted due to small number of studies. |
| Reporting biases | 21 | Present assessments of risk of bias due to missing results (arising from reporting biases) for each synthesis assessed. | 17, Supplementary 4 |
| Certainty of evidence | 22 | Present assessments of certainty (or confidence) in the body of evidence for each outcome assessed. | Evidence for each outcome group appraised in discussion. |
| **DISCUSSION** | | |  |
| Discussion | 23a | Provide a general interpretation of the results in the context of other evidence. | 22-26 |
|  | 23b | Discuss any limitations of the evidence included in the review. | 22-26 |
|  | 23c | Discuss any limitations of the review processes used. | 25-26 |
|  | 23d | Discuss implications of the results for practice, policy, and future research. | 22-26 (relevant information throughout discussion) |
| **OTHER INFORMATION** | | |  |
| Registration and protocol | 24a | Provide registration information for the review, including register name and registration number, or state that the review was not registered. | 6 |
|  | 24b | Indicate where the review protocol can be accessed, or state that a protocol was not prepared. | Prospectively registered on PROSPERO as above. No separate protocol published. |
|  | 24c | Describe and explain any amendments to information provided at registration or in the protocol. | Amendments to information provided at registration available on PROSPERO with accompanying justifications. |
| Support | 25 | Describe sources of financial or non-financial support for the review, and the role of the funders or sponsors in the review. | 27 |
| Competing interests | 26 | Declare any competing interests of review authors. | 27 |
| Availability of data, code and other materials | 27 | Report which of the following are publicly available and where they can be found: template data collection forms; data extracted from included studies; data used for all analyses; analytic code; any other materials used in the review. | 28 |
