# Supplementary material for: Impact of clinical decision support software on empirical antibiotic prescribing and patient outcomes: a systematic review and meta-analysis
Source: BMJ Open. 2025 Nov 27;15(11):e099100. doi: 10.1136/bmjopen-2025-099100 (PMC12666123; doi:10.1136/bmjopen-2025-099100)
Supplement: Supplementary file 2 [file bmjopen-2025-099100.reviewer_comments.pdf]

PEER REVIEW HISTORY

BMJ Open publishes all reviews undertaken for accepted manuscripts. Reviewers are asked to complete a checklist review form and are provided with free text boxes to elaborate on their assessment. These free text comments are reproduced below.

ARTICLE DETAILS

Title (Provisional)

The Impact of Clinical Decision Support Software on Empirical Antibiotic Prescribing & Patient Outcomes: A Systematic Review & Meta-analysis

Authors

Hatton, Christopher; Quarton, Samuel Jacob; Livesey, Alana; Alenazi, Bushra Ali; Jeff, Charlotte; Sapey, Elizabeth

---

VERSION 1 - REVIEW

**Reviewer**	**1**
**Name**	**Chootrakool, Hathaikan**
**Affiliation**	**Mahidol University, Biostatistics**
**Date**	**13-Feb-2025**
**COI**	**None**

The abstract is not very clear, especially the time scope in review (by February 2024). The authors could add more statistical analysis and show in abstract. Authors could create the funnel plot in different significant level.

**Reviewer**	**2**
**Name**	**Henley, Josie**
**Affiliation**	**Cardiff University, School of Social Sciences**
**Date**	**12-Jun-2025**
**COI**	**None**

The article needs a statistical review and I am not a statistician so cannot provide this, however from a lay reader's perspective the stats are understandable.

One thing that would be useful is a list of the names of the CDSS in the table of supplementary information 2. It is not entirely clear which of these CDSS are bespoke, developed for the intervention and which (if any) are commercially available and modified

for the intervention. More detail on this would be useful. For example, did multiple studies use the same CDSS but with slight modifications?

On the same note, in terms of the two key studies that are noted in the discussion (p.22), both by Gohil et al (references 28 and 29), it would be useful to have more information about the CDSS in these studies. I can surmise from the information in this article that both of these papers are from the same large study but targeting different infections. However it is not clear without finding the original papers whether everything else was consistent - i.e. same hospitals, same clinicians, same CDSS. It would be useful to know whether the CDSS was entirely the same, or whether the same but with different algorithms for the different infections, or whether different CDSS were used. Also, within the same large study it could be that different streams used a subset of sites. This would also be useful to know given the discrepancy between 28% reduction in prescribing for pneumonia and 17% for UTI - whether this was entirely due to the risks involved with infection, or the CDSS, or other factors.

**Reviewer**	**3**
**Name**	**Dack, Kyle**
**Affiliation**	**University of Bristol, MRC Integrative Epidemiology Unit**
**Date**	**29-Jun-2025**
**COI**	**None**

Statistical review summary:

The methodology is clear and transparent, but suggest adding subheadings to improve navigability. The statistical analysis uses reliable methods, and results are reported very clearly. My only statistical concern is that there is heterogeneity present in some of the meta-analysis results, but this is not explored through subgroup analyses. It may be there are a lack of studies to perform a full quantitative subgroup analysis, but there could still be qualitative comparisons between groups.

Abstract:

1. I suggest either in the objectives or methods subheading to add clarification of what patient/clinical outcomes are of interest, because this could include quite include quite a broad range of measures.

2. What was the association and uncertainty estimate regarding antimicrobial stewardship and unnecessary prescriptions? I suggest reporting these, for consistency with the other reported results.

Strengths and limitations:

3. The third point states that the study was limited by most studies being low quality. This may be true but it seems inconsistent with the statement in the abstract that there was high

quality evidence for two of the outcomes. I tried to view the quality of studies to understand this, but it is quite challenging because a summary table or figure of study quality is not reported. I can see the full details are available in Table S3, but a summary would help readers. The ROBINS documentation gives an example of this: https://methods.cochrane.org/sites/methods.cochrane.org/files/uploads/inline-files/Final_ROBINS-I%20author%20guidance_0.pdf (page 9).

Methods:

4. I suggest adding subheadings matching the PRISMA subsections to help readers navigate this.

5. The second paragraph defines the intervention, and reports the inclusion criteria. I think this would be clearer if separated out with one section solely for inclusion/exclusion criteria, and a separate section for explaining the intervention, and defining outcomes (which correspond to prisma sections 8 and 10).

6. Similar to the above I suggest dividing paragraph 3 which currently reports data extraction and outcome definitions and RoB assessment. Separate paragraphs and/or subheadings would improve clarity.

7. There is some important information missing regarding the outcomes, which impacts how the results can be understood. "Outcome data were extracted and grouped into four broad categories including clinical outcomes" which measures were specified for this? How were these quantified – e.g. odds ratios, or units of analysis. Additionally, how were results grouped or screened – e.g. if multiple timepoints were reported which were selected, or what time ranges were desired? Finally were any transformations applied to ensure outcome estimates were compatible? I suggest adding this information for all outcomes, so that readers understand exactly what results to expect.

8. "Therefore, studies or outcomes that reflected the impact of adherence to CDSS recommendations were excluded" this is more suitable to be in the exclusion criteria rather than near the statistical analysis.

9. "Outcomes with sufficient reporting and homogeneity were synthesised quantitatively" I agree that these are reasonable criteria and often involve an element of judgement, but were there any specific thresholds used?

10. My primary concern regarding the methodology and statistical analysis is that it does not contain an approach to exploring heterogeneity. If heterogeneity is high, a random effects model may accommodate it, but it does not really help explain it. For this, it is recommended (e.g. in the Cochrane handbook) that subgroup analyses are performed to find groupings with less heterogeneity. Either quantitatively, or if there are a lack of studies, qualitatively. For example my measurement methods, populations, or quality.

11. Certainty of evidence assessment was part of the analysis, this should be reported in the methods.

Results

12. Study & Intervention Characteristic: The line spacing varies in this section.

13. “There was variable and incomplete adjustment for potential confounding across all non-randomised studies” is it possible to report more details on this – in particular were there any critical confounders which studies missed?

14. There was moderately high heterogeneity reported for the meta-analysis of mortality. The authors report that there were some differences between studies, e.g. in the definition of mortality, confounder adjustment, and study quality. When looking at these differences, are there any patterns which may explain the high heterogeneity? This should be explored and reported because the main pooled estimate is not fully valid for all studies/populations, and finding why this is would be very valuable.

15. Other clinical outcomes lacked adequate numbers of studies or had too high heterogeneity. I agree with the authors compromise of summarising results from these studies qualitatively.

16. I suggest removing the Q statistic from the reports of heterogeneity because it is not directly interpretable by readers, and the I2 and p-values will give enough information.

Discussion

17. The discussion provides a good summary, including interpretation and certainty. What I think should be emphasized a little more and perhaps discussed is the heterogeneity in some of the results. As mentioned, it does impact the validity of the results, which readers should be reminded of. It would also be informative if possible reasons were given for the heterogeneity.

**Reviewer**	**4**
**Name**	**Sunderland, Bruce**
**Affiliation**	**Curtin University, School of Pharmacy**
**Date**	**28-Jul-2025**
**COI**	**None**

This systematic review has been well conducted and every aspect of it is clearly described. It does suffer from the low-quality studies available to review and their heterogeneous analyses. Systems that enhance prescribing and patient outcomes from antibiotics are important as inappropriate prescribing is an important factor in the fight against escalating resistance. The Abstract appears quite long, however, only provides two outcomes. Both the low quality of the studies available and additional findings should be included.

The Background is adequate for the review, but requires many of the assertions made to be given appropriate literature references, Methods: It is unclear whether patients admitted or

presenting at hospital already prescribed antibiotics were included or excluded. Lines 47 to 54 (page 8) should be further explained why CDSS implementation is less prone to bias then Adherence to CDSS Recommendations, Notably in Lines 10 Page 8 antibiotic appropriateness is an outcome of the review.

Results: The section from Line 54 on Page 10 to Line 22 on Page 11 is mainly discussion. It is notable there is a wide range of publication dates from 1994 to 2024. Several were published more than a decade ago. As these systems are usually computer based and benefit from being linked to a patient electronic record, this is less likely for earlier versus recent publications. The authors indicate all studies were non-randomised which is correct. Before-after or before-after with a control group are common methodologies for such interventions in a hospital setting. A large amount of detailed material is provided in the supplementary materials which will be of value to a wider range of readers. The Discussion is detailed and covers the relevant sections of the review. The methodology criteria have narrowed the review to a small number of studies. The Conclusions could be slightly expanded.

VERSION 1 - AUTHOR RESPONSE

Reviewer 1 Comments

Comment 1: The abstract is not very clear, especially the time scope in review (by February 2024). The authors could add more statistical analysis and show in abstract.

Response: The abstract has been comprehensively re-written and re-structured to reflect this feedback.

Comment 2: Authors could create the funnel plot in different significant level.

Response: The funnel plot has been revised and includes 90%, 95%, and 99% significance levels.

Reviewer 2 Comments

Comment 1: One thing that would be useful is a list of the names of the CDSS in the table of supplementary information 2. It is not entirely clear which of these CDSS are bespoke, developed for the intervention and which (if any) are commercially available and modified for the intervention. More detail on this would be useful. For example, did multiple studies use the same CDSS but with slight modifications?

Response: This information has now been added to Supplementary 2 so that readers can easily see where different studies have evaluated the impact of the same CDSS system. Given the small number of studies, it is difficult to establish comparative effectiveness, as discussed with heterogeneity in the discussion.

Comment 2: On the same note, in terms of the two key studies that are noted in the discussion (p.22), both by Gohil et al (references 28 and 29), it would be useful to have more information

about the CDSS in these studies. I can surmise from the information in this article that both of these papers are from the same large study but targeting different infections. However it is not clear without finding the original papers whether everything else was consistent - i.e. same hospitals, same clinicians, same CDSS. It would be useful to know whether the CDSS was entirely the same, or whether the same but with different algorithms for the different infections, or whether different CDSS were used. Also, within the same large study it could be that different streams used a subset of sites. This would also be useful to know given the discrepancy between 28% reduction in prescribing for pneumonia and 17% for UTI - whether this was entirely due to the risks involved with infection, or the CDSS, or other factors.

Response 2: The discussion has been expanded to include a clearer description of these studies, with some brief discussion about potential reasons for these discrepancies:

"Notably, two of these studies were at low risk of bias. These studies by Gohil and colleagues were cluster RCTs that randomised over 50 hospitals to the implementation of CDSS for antimicrobial stewardship. The studies were conducted concurrently over the same period and in the same hospitals. The CDSS provided an antimicrobial stewardship prompt for patients at low risk of infection secondary to a multi drug resistant organism (MDRO) who were about to be prescribed an extended spectrum antibiotic. The risk of MDRO was calculated using a classification and regression tree algorithm using retrospective data separately for pneumonia (30) and UTI (31), and different individual and organisational factors were associated with MDROs in each condition. The introduction of these prompts was associated with a relative reduction in broad spectrum antibiotic prescribing of 28% in pneumonia and 17% in UTI. The baseline prescribing rate of broad spectrum antibiotics was greater in the pneumonia cohort, which could partly account for the discrepancy in the effectiveness of these prompts, although there are also other plausible explanations."

Reviewer 3 Comments

Comment 1: I suggest either in the objectives or methods subheading to add clarification of what patient/clinical outcomes are of interest, because this could include quite include quite a broad range of measures.

Response: The abstract has been re-structured, and the specific clinical outcomes reported are included.

"Outcomes were grouped into four domains including clinical outcomes (mortality, length of stay, readmission rates), antibiotic appropriateness (guideline adherence, coverage of causative organism) …"

Comment 2: What was the association and uncertainty estimate regarding antimicrobial stewardship and unnecessary prescriptions? I suggest reporting these, for consistency with the other reported results.

Response: Quantitative synthesis was not performed for antimicrobial stewardship outcomes and therefore there is no uncertainty estimate to report for this domain.

Comment 3: Strength and Limitations - The third point states that the study was limited by most studies being low quality. This may be true but it seems inconsistent with the statement in the abstract that there was high quality evidence for two of the outcomes. I tried to view the quality of studies to understand this, but it is quite challenging because a summary table or figure of study quality is not reported. I can see the full details are available in Table S3, but a summary would help readers. The ROBINS documentation gives an example of this: https://methods.cochrane.org/sites/methods.cochrane.org/files/uploads/inline-files/Final_ROBINS-I%20author%20guidance_0.pdf (page 9).

Response: The strengths and limitations have been re-written because only one sentence for each was permitted as per BMJ Open formatting. The revised strengths and limitations avoid this confusion:

- This systematic review identified high quality evidence which suggests that the use of Clinical Decision Support Software (CDSS) can positively influence antimicrobial stewardship in hospital.
- There was substantial heterogeneity in the results of individual studies, and it was difficult to understand the reasons for this variance given the small number of included studies.

Summary figures of risk of bias have been added to Supplementary 3 for each outcome domain as suggested.

Comment 4: I suggest adding subheadings matching the PRISMA subsections to help readers navigate this.

Response: Subheadings matching PRISMA subsections have now been added to improve navigability of the methods section.

Comment 5: The second paragraph defines the intervention, and reports the inclusion criteria. I think this would be clearer if separated out with one section solely for inclusion/exclusion criteria, and a separate section for explaining the intervention, and defining outcomes (which correspond to prisma sections 8 and 10).

Response: These sections have been separated into different paragraphs to improve clarity. We felt that the operational definition used for CDSS was an important aspect of study eligibility, so have opted to describe this under this subheading but as a separate paragraph for clarity.

Comment 6: Similar to the above I suggest dividing paragraph 3 which currently reports data extraction and outcome definitions and RoB assessment. Separate paragraphs and/or subheadings would improve clarity.

Response: The methods section has been re-structured to improve navigability and these are all now discussed under separate subheadings.

Comment 7: There is some important information missing regarding the outcomes, which

impacts how the results can be understood. “Outcome data were extracted and grouped into four broad categories including clinical outcomes” which measures were specified for this? How were these quantified – e.g. odds ratios, or units of analysis. Additionally, how were results grouped or screened – e.g. if multiple timepoints were reported which were selected, or what time ranges were desired? Finally were any transformations applied to ensure outcome estimates were compatible? I suggest adding this information for all outcomes, so that readers understand exactly what results to expect.

Response: This section has been expanded to reflect these suggestions.

The specific clinical outcomes that were extracted have been clarified: “Clinical outcomes included mortality, length of hospital stay, and readmission to hospital.”

Further detail has been provided about the time points extracted: “Given the sparsity of evidence, no pre-specified time range of interest was defined and all analyses relevant to the outcomes were extracted. Studies rarely reported multiple time points, but where this occurred short term outcomes (i.e. within 30 days or less) were prioritised for inclusion in quantitative synthesis, as they were considered more likely to be related to the intervention than longer term outcomes. Some studies reported multiple intervention phases, often including a phase where CDSS was implemented alone, and a phase where it was implemented with other antimicrobial stewardship interventions. In these studies, the intervention phase that reflected the implementation of CDSS with the fewest co-interventions was used as the outcome, and the phase with fewest interventions was used as the baseline comparator.”

Relevant information about the extraction of outcome estimates has been provided: “All outcomes that were synthesised quantitatively were dichotomous, and were either extracted as ORs or converted to ORs using raw values, p-values, or 95% confidence intervals of included studies using the *metafor* package. Where adjusted and unadjusted results were reported, adjusted results were extracted. Confounding bias is common in studies evaluating complex interventions, particularly non-randomised studies, and therefore adjusted results were thought to be more representative of the true effect of the intervention.”

Comment 8: “Therefore, studies or outcomes that reflected the impact of adherence to CDSS recommendations were excluded” this is more suitable to be in the exclusion criteria rather than near the statistical analysis.

Response: This section has been moved under the eligibility criteria subheading.

Comment 9: “Outcomes with sufficient reporting and homogeneity were synthesised quantitatively” I agree that these are reasonable criteria and often involve an element of judgement, but were there any specific thresholds used?

Response: Pre-specified thresholds were not used to make this decision, but we have described the approach taken more clearly in the text to help reader’s understand how this decision was made.

“Outcomes with sufficient reporting and homogeneity were synthesised quantitatively using a random-effects meta-analysis with the *metafor* package (19) in R Version 4.3.0 (20) using the

restricted maximum likelihood (REML) method. Decisions surrounding whether to perform quantitative synthesis for individual outcomes was made qualitatively, rather than using pre-specified thresholds. Generally, outcomes were quantitively synthesised if they were reported consistently or could be converted to a consistent measure across most studies."

Comment 10: My primary concern regarding the methodology and statistical analysis is that it does not contain an approach to exploring heterogeneity. If heterogeneity is high, a random effects model may accommodate it, but it does not really help explain it. For this, it is recommended (e.g. in the Cochrane handbook) that subgroup analyses are performed to find groupings with less heterogeneity. Either quantitatively, or if there are a lack of studies, qualitatively. For example my measurement methods, populations, or quality.

Response: As mentioned, the small number of studies reporting separate outcomes does limit the extent to which we can understand the heterogeneity observed results. However, both the results and discussion section have been comprehensively revised to address this as far as possible, and this has been discussed under the limitations section also.

Results section: "A substantial proportion of heterogeneity in mortality outcomes arose from a single study, which observed an increased death rate during the CDSS period. However, this result did not adjust for important observed differences in case-mix, which may account for this discrepant finding. Other sources of heterogeneity include differences in study setting, study design, infectious syndrome, CDSS characteristics, and outcome measures used. There was no clear and consistent relationship between these features and study results."

Results section: "Guideline adherent antibiotic prescribing improved following the implementation of CDSS (OR 1.75, 95% CI 1.26 – 2.43, $p < 0.001$). There was substantial heterogeneity associated with this result ($I^2 = 59.9\%$, $p = 0.07$). This heterogeneity may have been related to the infectious syndrome the CDSS was used for. The results of three studies that explored the impact of CDSS implementation in pneumonia or skin and soft tissue infection identified a consistent improvement in guideline adherence of similar magnitude. However, the results of the one study that explored the use of CDSS for antibiotic prescribing in UTI did not identify any improvements in guideline adherent prescribing. There are also several other sources of heterogeneity including differences in study design, baseline guideline adherence, CDSS content, and setting that may have also accounted for some of the differences observed."

Limitations in discussion: "Heterogeneity was observed in the outcomes of included studies across most outcome domains. There were several potential sources of heterogeneity including study design, setting, organisational context, time of implementation, statistical analysis, characteristics of the intervention, and infectious syndrome. The small number of studies made it difficult to fully understand the cause of this heterogeneity and whether certain characteristics of CDSS were associated with effectiveness. Substantial heterogeneity has been observed in prior systematic reviews and meta-analyses exploring the effectiveness of CDSS more broadly (53) and it is uncertain how CDSS can be designed with the best chance of success."

Comment 11: Certainty of evidence assessment was part of the analysis, this should be reported in the methods.

Response: This has been specified in the methods section: “The certainty of evidence for each outcome domain was evaluated qualitatively based on the risk of bias assessments, together with the consistency, directness, and precision of results.”

Comment 12: Study & Intervention Characteristic: The line spacing varies in this section.

Response: This has been resolved

Comment 13: “There was variable and incomplete adjustment for potential confounding across all non-randomised studies” is it possible to report more details on this – in particular were there any critical confounders which studies missed?

Response: Specific sources of bias have now been included explicitly in this statement.

“There was variable and incomplete adjustment for potential confounding including disease severity, case-mix, and temporal trends across all non-randomised studies.”

Comment 14: There was moderately high heterogeneity reported for the meta-analysis of mortality. The authors report that there were some differences between studies, e.g. in the definition of mortality, confounder adjustment, and study quality. When looking at these differences, are there any patterns which may explain the high heterogeneity? This should be explored and reported because the main pooled estimate is not fully valid for all studies/populations, and finding why this is would be very valuable.

Response: This has been addressed in the response to comment 10 which included discussion about the heterogeneity observed for mortality, and more broadly amongst other outcomes.

Comment 15: I suggest removing the Q statistic from the reports of heterogeneity because it is not directly interpretable by readers, and the I2 and p-values will give enough information.

Response: This has been removed from the in-text reports of heterogeneity.

Comment 16: The discussion provides a good summary, including interpretation and certainty. What I think should be emphasized a little more and perhaps discussed is the heterogeneity in some of the results. As mentioned, it does impact the validity of the results, which readers should be reminded of. It would also be informative if possible reasons were given for the heterogeneity.

Response: In addition to the discussion of heterogeneity in the results section, it has also been highlighted in the limitations section of the discussion and conclusion.

“Heterogeneity was observed in the outcomes of included studies across most outcome domains. There were several potential sources of heterogeneity including study design, setting, organisational context, time of implementation, statistical analysis, characteristics of the

intervention, and infectious syndrome. The small number of studies made it difficult to fully understand the cause of this heterogeneity and whether certain characteristics of CDSS were associated with effectiveness. Substantial heterogeneity has been observed in prior systematic reviews and meta-analyses exploring the effectiveness of CDSS more broadly (53) and it is uncertain how CDSS can be designed with the best chance of success."

Reviewer 4

Comment 1: The Abstract appears quite long, however, only provides two outcomes. Both the low quality of the studies available and additional findings should be included.

Response: The abstract has been re-structured and re-written to make it clearer and more comprehensive for readers.

Comment 2: The Background is adequate for the review, but requires many of the assertions made to be given appropriate literature references

Response: Further literature references have been added to support assertions in the background where relevant.

Comment 3: It is unclear whether patients admitted or presenting at hospital already prescribed antibiotics were included or excluded.

Response: It is likely that some studies included patients that had been prescribed antibiotics in the community prior to presentation or admission to hospital. However, this was not reported explicitly by most included studies. A statement has been added to the methods section to make this clear:

"Whilst the primary aim was to explore initial empirical prescribing it is possible that some patients included in studies had antibiotics prescribed in the community prior to attendance at hospital."

Comment 4: Lines 47 to 54 (page 8) should be further explained why CDSS implementation is less prone to bias then Adherence to CDSS Recommendations, Notably in Lines 10 Page 8 antibiotic appropriateness is an outcome of the review.

Response: In clinical practice there are likely to be non-random reasons for non-adherence to CDSS recommendations. For example, non-adherence may occur because there is diagnostic uncertainty about the source of infection, or because they have recent sensitivity results showing resistance to a possible organism. Therefore, comparing outcomes between these two groups is likely to be biased, and difficult to adjust for either through study design or statistical adjustment. A statement has been added to clarify the rationale for this decision:

"This decision was made because results from analyses comparing adherence to non-adherence are likely to be prone to significant bias; there are likely to be reasons for non-adherence in many cases resulting in non-comparable populations."

Comment 5: The section from Line 54 on Page 10 to Line 22 on Page 11 is mainly discussion.

Response: This section does have the feel of discussion but is recommended for inclusion in this results section by PRISMA guidelines. For consistency, we have kept this section in the results section.

Comment 6: It is notable there is a wide range of publication dates from 1994 to 2024. Several were published more than a decade ago. As these systems are usually computer based and benefit from being linked to a patient electronic record, this is less likely for earlier versus recent publications.

Response: Due to the small number of studies exploring each outcome domain it is difficult to assess whether the heterogeneity in outcomes is related to time of CDSS implementation. Qualitatively, it does not appear that outcomes are related to time of implementation. We have added comprehensive discussion about heterogeneity to the results and discussion section.

Comment 7: The Conclusions could be slightly expanded.

Response: The conclusion section has been expanded to reflect this feedback.

"The implementation of CDSS to guide empirical antibiotic prescribing in hospital can positively influence the prescribing of antibiotics, improve guideline adherence, and reduce the prescribing of broad-spectrum antibiotics. Despite this, it is uncertain whether these changes translate to clinical benefit, predominantly because of limitations in study design resulting in high risks of bias. Further work is required to understand the variation in outcomes that result from CDSS implementation, to ensure that future systems are optimised."
